# Host-membrane lipid composition controls *Cryptococcus neoformans* cellular targets

**DOI:** 10.3389/fimmu.2025.1687068

**Published:** 2026-01-06

**Authors:** Aswathi I. Ramesh, Sumukha Hegde, Aarsh Mahendra Dabhi, Sriram Krishnamurthy, Divya Chandran, Shivakumar K. Reddy, Prakash Peralam Yegneswaran, Kavitha Saravu, P.V. Mallikarjuna, Kavitha S. Shettigar, Pritesh Bhat, Varadaraj Bhat, K.R. Sabitha, Indumathi Mariappan, Vishukumar Aimanianda, Ashok K. Shetty, Dinesh Upadhya

**Affiliations:** 1 Centre for Molecular Neurosciences, Kasturba Medical College Manipal, Manipal Academy of Higher Education, Manipal, India; 2 Schrodinger India Private Limited, Hyderabad, India; 3 Department of Microbiology, Kasturba Medical College, Manipal Academy of Higher Education, Manipal, India; 4 Department of Infectious Diseases, Kasturba Medical College Manipal, Manipal Academy of Higher Education, Manipal, India; 5 Department of Medical Laboratory Technology, Manipal College of Health Professions, Manipal Academy of Higher Education, Manipal, India; 6 Schrodinger India Private Limited, Bengaluru, India; 7 Department of Pharmaceutical Chemistry, Manipal College of Pharmaceutical Sciences, Manipal Academy of Higher Education, Manipal, India; 8 Center for Ocular Regeneration (CORE), Prof. Brien Holden Eye Research Center (BHERC), L.V. Prasad Eye Institute, Hyderabad, India; 9 BRIC- inStem, Bangalore, India; 10 Department of Biochemistry, Kasturba Medical College Manipal, Manipal Academy of Higher Education, Manipal, India; 11 Institute for Regenerative Medicine, Department of Cell Biology and Genetics, Naresh Vashisht College of Medicine, Texas A&M University Health Science Center, College Station, TX, United States

**Keywords:** cerebral organoids, *Cryptococcus neoformans*, meningoencephalitis, pluripotent stem cells, human neurons, membrane lipids, neural stem cells, lipidomics

## Abstract

**Background:**

*Cryptococcus neoformans* causes lethal meningoencephalitis and long-term neurological deficits, particularly in immunocompromised hosts of any age group. After penetrating the airway and crossing the blood-tissue barriers, *C. neoformans* rapidly enters the brain, where it extensively releases the capsular polysaccharide glucuronoxylomannan (GXM), a major virulence factor.

**Methods:**

*Cryptococcus neoformans (*ATCC 32045) was used to isolate and purify GXM. This study utilized human-induced pluripotent stem cell-derived 2D cultured neural stem cells, neurons, and microglia-deficient cerebral organoids to identify GXM-induced pathogenesis. Cryosectioning of frozen tissues, immunostaining, western blot analysis and untargeted lipidomics were employed to identify the GXM-induced impact on cellular proliferation and cell death, as well as possible cellular and molecular targets. The study utilized the first-ever atomistic models of neuronal and neural stem cells (NSC) membranes, built using a proportion of the original lipid compositions using the Materials Science Suite to identify subtle interactions between the membranes and GXM.

**Results:**

GXM exposure induced subtle cell death, but progenitor cell proliferation was unaffected. Interestingly, GXM preferentially targeted neurons irrespective of the abundance of NSCs and astrocytes. Synaptophysin, an integral component of neuronal synaptic vesicles, was significantly reduced following GXM exposure. The untargeted lipidomics analysis revealed higher phosphatidylcholine levels and reduced phosphatidylethanolamine levels in human neurons compared to other cell types. The atomistic models revealed a significant attractive interaction energy between GXM and neuronal membranes, with phosphatidylcholine being primarily responsible.

**Conclusion:**

This study provides novel evidence that lipid membranes containing higher phosphatidylcholine are a primary target of GXM of *C. neoformans* and could be the possible reason for the preferential targeting of GXM to neurons. Additionally, GXM induced synaptic deficits in neurons, which could be a significant factor contributing to the neurological dysfunctions observed in this fungal infection. This study opens the mechanism of pathogenesis and targeting opportunities for treating *C. neoformans-*induced meningoencephalitis.

## Introduction

*Cryptococcus* genus consists of two major pathogenic species of encapsulated yeast, *C. neoformans* and *C. gatti*, which are known to cause life-threatening meningoencephalitis in humans, particularly in immunocompromised hosts of any age group (1–5). According to the Fungal Priority Pathogen List (FPPL) published by the World Health Organization (WHO), *C. neoformans* is one of the critically risky pathogens, while *C*. *gattii* is a medium-priority pathogen (6). Globally, the incidence of cryptococcal meningitis accounts for nearly 152,000 cases each year, with around 112,000 deaths (7). Focal neurological symptoms are a significant predictor of mortality at 2 and 10 weeks post-infection (8). Alarmingly, 20-70% of survivors of *C. neoformans* infection worldwide have long-term neurological deficits at one year (9).

*C. neoformans* is an airborne pathogen. Under immunocompromised conditions, it can invade tissues quickly and manifest pathogenesis; in immunocompetent conditions, it can stay dormant, dramatically escaping the host immune cell vigilance for years before manifesting the symptoms under favorable conditions (10). Upon revival and pathogenesis, the most severe illness is caused by the migration of this pathogen to the central nervous system. After breaching the airway and blood tissue barriers, *C. neoformans* rapidly invades the brain, where it is engulfed by microglia (11). Studies demonstrate that microglia are nonprotective and act as reservoirs for *C. neoformans* (12). *C. neoformans* infection is also shown to cause morphological changes in host microglia, leading to their dysfunction (13).

During pathogenesis, *C. neoformans* extensively releases glucuronoxylomannan (GXM), its capsular polysaccharide and a major virulent factor that facilitates host cellular entry of this pathogen (14, 15). GXM is known to cross the blood-brain barrier and accumulate throughout the parenchyma in areas surrounding the infected tissues (15–21). Cryptococcal polysaccharide is present persistently in tissues at low quantities months after administration in immunodeficient animals (22), and continued presence in the brain at low quantities even after antifungal therapy could be a reason for the long-term complications of cryptococcal infection (23, 24). The presence of GXM in the brain further enhances the invasion of *C. neoformans* into the brain, leading to defective immunity and microglial dysregulation (13, 21). Meanwhile, microglia migrate away from the sites of GXM accumulation (13).

The question is then, how does GXM affect other brain cells and cause long-lasting pathologies associated with *C. neoformans* meningoencephalitis?

To fully understand host-pathogen interactions, several researchers have utilized cerebral organoid models (25–29). With undirected differentiation, cerebral organoids contain neural stem cells, neurons, and astroglia cells, while microglia are mostly lacking (30). The modified protocols could generate organoids with innate microglia (31). The study utilized up to day-60 organoids as they provide an ideal opportunity to study the targets of GXM action in the presence of abundant neurons, astroglia, and neural stem cells in a unique environment without microglia. In this study, 2D cultured human neural stem cells, neurons, and cerebral organoids were utilized to decipher their interaction with GXM. Moreover, the study utilized the first-ever atomistic models of neuronal and NSC membranes, built using a proportion of the original lipid compositions for identifying subtle interactions between the neuronal membranes and GXM.

## Materials and methods

Details of the chemicals used, antibody sources, and fungal strains used are provided in **Supplementary Table S1**.

### Fungal strain

*Cryptococcus neoformans* ATCC 32045, grown in Yeast Peptone Dextrose medium at a pH of 6.5 at 30°C at 150 rpm, was used in this study.

### Glucuronoxylomannan isolation and purification

GXM isolation was carried out using methods previously described (32, 33). *C. neoformans* were cultured in 200 ml of capsule induction media containing a 1:10 dilution of Yeast Peptone Dextrose and phosphate-buffered saline (PBS)) for 5 days at 30°C with continuous shaking at 150 rpm. After 5 days of culture, the supernatant was retrieved by centrifugation at 10,000 rpm for 1 hour at room temperature. The supernatant was adjusted to 10% sodium acetate, and pH was maintained at 7.0 by adding acetic acid. Three volumes of ethanol were added to the supernatant to precipitate the capsular polysaccharide (PS) and incubated at 4°C overnight. PS collected after centrifugation was air-dried, and the total mass was estimated. To separate GXM from PS, the pellet was dissolved in 0.2M sodium chloride (NaCl, 10 mg/ml) overnight at 4°C, and Cetyl-trimethyl ammonium bromide (CTAB); 3 mg/mg of PS, was added slowly with continuous stirring at room temperature; 0.05% CTAB was added drop by drop to precipitate the CTAB-GXM complex. The solution was then centrifuged, and the precipitate was dissolved in NaCl (1M) overnight at 4°C. To precipitate GXM, 3 volumes of ethanol were added to the solution, and the precipitated GXM was recovered by centrifugation, air-dried at room temperature, dissolved in 2 M NaCl, and dialyzed against 1 M NaCl for 1 day and then against distilled water for 6 days. The dialysate was passed through a 0.22 µm filter, lyophilized, and stored. This prepared GXM was confirmed to be endotoxin-free using an endotoxin detection kit gel clot (Himedia, with a sensitivity of 0.03 EU/mL). Details of the resources are provided in **Supplementary Table S1**.

### Cerebral organoid culture

The cerebral organoid culture was performed according to the Lancaster and Knoblich (30) protocol. Briefly, human induced pluripotent stem cells (hiPSCs) (ND1.4, RRID: CVCL_1E77) were grown in StemFlex medium (Gibco Cat. no A3349401) and lifted off when 60-70% confluent to develop embryoid bodies (EBs). When EB’s have smooth edges with the formation of neuroepithelial cells, usually after 7 days of neural induction, they were embedded in a matrigel droplet and provided with cerebral organoid differentiation media containing 50% Dulbecco’s Modified Eagle Medium/F12 (DMEM/F12), 50% Neurobasal Media, 0.5% N2 supplement, 0.5% Non-Essential Amino Acid (NEAA), 1% Pencillin-streptomycin, 1% B27 without retinoic acid, 1% Glutamax supplement, 0.025% Insulin, and 0.035% β mercaptoethanol (1:100 dilutions of 2-Mercaptoethanol in DMEM-F12). The static culture was maintained for 4 days to facilitate the development of neuroepithelial buds, and thereafter, the cells were subjected to continuous orbital shaking for up to 30 days and 60 days to promote the growth of cerebral organoids. The cerebral organoid differentiation media contained 1% B27 with retinoic acid. These cerebral organoids were randomly divided into control and treatment groups with 6–7 organoids in each group (n=3). GXM (50 µg/ml) was dissolved in cerebral organoid differentiation media and added to the treatment group; only media was added to the control group. The organoids were returned to the shaker in the incubator for 48 hours at 37°C and 5% CO_2._

After 48 hours of GXM treatment, cerebral organoids from both the control and treatment groups were washed with 1X PBS and fixed in 4% paraformaldehyde (PFA) for 15 minutes. Following the removal of PFA, the organoids were washed with 1X PBS for 30 minutes and then incubated in a 30% sucrose solution overnight at 4°C. The next day, cerebral organoids were placed in a mold containing gelatin (7.5%)–sucrose (10%) solution in 1X PBS and snap-frozen by adding dry ice in isopentane solution by bringing the temperature to -30°C to -50°C. Frozen blocks were stored at -80°C until they were cryosectioned. Ten- micrometer cryosections were taken using an Epredia Microm HM525 NX Cryostat, and the sections were placed on gelatin-coated slides. These cryosections were stored at -20°C until staining for immunofluorescence studies.

### Neural stem cells culture

The hiPSCs were passed on Matrigel-coated 12-well plates in StemFlex medium (Gibco Cat. A3349401). When the cells reached 15-20% confluence, they were supplemented with Gibco PSC Neural Induction Media, which contains neurobasal medium and neural induction supplement (Cat. no. A1647801). On day 7 of neural induction, NSCs (P0) were ready for further expansion. These NSCs were dissociated, passaged to a new matrigel-coated well, and supplemented with Neural Expansion media (50% Neurobasal medium, 50% DMEM/F12, 2% Neural Induction supplement). Media changes were performed every other day until the NSCs were confluent. These NSCs were cultured on a coverslip coated with Matrigel to study cell proliferation. After 48 hours of seeding, three different concentrations of GXM (50-100 µg/ml) were dissolved in the neural expansion media and supplemented to the cells. A well without GXM treatment was taken as standard control. After 48 hours of GXM treatment, immunostaining was performed using the Ki67 antibody. Images were taken at 40x magnification in different fields from each group. Ki-67-positive cells and DAPI-positive cells in each image from 20 different fields were counted manually, and the percentage of cell proliferation was calculated.

### Differentiation of expanded NSCs to neuronal culture

For neuronal culture, NSCs were dissociated and plated on a Poly-L-Ornithine (overnight)/Laminin (1 hour) coated well and supplemented with Neural differentiation medium (Neurobasal medium, 1% N2, 2% B27 with retinoid acid, Brain-derived neurotrophic factor (BDNF) (2.5 ng/ml), Glial cell line-derived neurotrophic factor (GDNF) (1 ng/ml) as described earlier (34). The media was changed every other day, and the neuronal cells were grown for up to 21 days, confirmed by the cell-specific marker TUJ1. To examine the expression of synaptophysin, the neurons were treated with 50 µg/ml of GXM. Three different concentrations of GXM (50-100 µg/ml) were used to study cellular apoptosis. GXM treatment was administered for 48 hours, after which the neuronal cells were dissociated and subjected to western blot analysis.

### TUNEL assay

Terminal deoxynucleotidyl transferase-mediated dUTP nick end labeling (TUNEL) assays were done using TAKARA *in situ* Apoptosis Detection Kits (TAKARA Cat. no MK 500), according to the manufacturer’s instructions as described earlier (35). Briefly, cerebral organoid sections were treated with a permeabilization buffer for 5 minutes and sequentially incubated with a mixture of labelling-safe buffer and TdT enzyme for 90 minutes at 37°C. After 90 min, the sections were washed using 1X PBS and counterstained with DAPI (1:300).

### Immunofluorescence

For immunofluorescence staining, slides with sections bordered using a PAP pen were fixed in 4% PFA for 15 minutes. After fixation, the sections were washed using 1X PBS for 15 min. Permeabilization and nonspecific blocking were done using a solution containing 1X PBS, 0.2% Triton X-100, and 10% Donkey serum for 1 hour at room temperature. Primary antibodies such as NESTIN, SOX2, βIII tubulin, GFAP, Ki67, Synaptophysin, anti-GXM antibody were diluted in 1X PBS along with 0.2% Triton X-100 (v/v) and 5% Donkey serum and kept overnight at 4°C.

Excess primary antibodies were washed using 1X PBS. Then sections were incubated with specific secondary antibodies such as Alexa Flour 488 donkey anti-rabbit (1:500), Alexa Flour 594 donkey anti-mouse (1:500), Alexa Flour 488 donkey anti-mouse (1:500) Alexa Flour 568 donkey anti-rabbit (1:500) diluted in 1 X PBS along with 0.2% Triton X-100 (v/v) and 5% donkey serum for 1 hour at room temperature. Later, the secondary antibody was removed, and the sample was washed with 1X PBS. DAPI (1:300) was added, incubated for 10 minutes, and washed with 1X PBS for nuclear staining. The slides were then mounted and observed under a fluorescent microscope. Images were taken using the Cell Sens software at different magnifications. For counting Ki-67 and TUNEL-positive cells, 15 images from different areas in each group, taken at 40X magnification, were manually selected and counted. Cerebral organoid sections were imaged as Z stacks using a Leica Stellaris 5 confocal microscope using a 63x objective.

### Western blot analysis

Neurons were dissociated 48 hours post-GXM treatment and lysed using a radioimmunoprecipitation assay (RIPA) mixture supplemented with a protease inhibitor. The total protein concentration in each group was estimated using the Bicinchoninic acid assay (BCA) protein assay kit. Using sodium dodecyl sulfate-polyacrylamide gel electrophoresis, an equal amount of protein was separated from the control and GXM-treated group and transferred to a polyvinylidene difluoride membrane (PVDF). Anti-synaptophysin (1:5000) and beta-actin (1:5000) primary antibodies were used. Following imaging, the chemiluminescence method and band intensity were analyzed using ImageJ software.

### Isolation of lipids from neural stem cells and neurons

Lipids were extracted using a slightly modified Bligh & Dyer protocol (36) and described in detail in our earlier paper (37). NSCs and isolated neurons were reconstituted in 500 µl of 1x PBS + 1% protease inhibitors, and a small 50 µl aliquot was used for total protein quantification by the BCA method. Briefly, 450 µL of samples were added to 1.875 mL of a 1:2 (v/v) chloroform: methanol mixture, vortexed, and then sonicated for 1 minute. The mixture was then added to chloroform and distilled water, vortexed for 30 minutes, and centrifuged at 5000 rpm at 4°C for 15 minutes to facilitate phase separation. The aqueous phase and protein interphase were discarded, and the organic (lipids) phase was transferred to a new vial. The organic phase was washed again with a chloroform: methanol: water mixture, as described in the protocol. The final organic phase was then air-dried under a nitrogen stream, and subsequently, the lipids were dissolved in 100 µL of a 2:1 (v/v) chloroform: methanol solution in a glass vial.

### LC-MS/MS untargeted method

Samples were analyzed using Thermo Q-Exactive coupled to a Dionex UPLC chromatographic system of the LC-MS model. All the samples were analyzed in triplicate in both positive and negative modes. A total of 5 µl was injected into the Acclaim C30 column at a flow rate of 300 µl/min. The chromatographic separation was performed using ammonium formate with formic acid in a gradient of acetonitrile and water/water and ammonium formate with formic acid in a gradient of isopropanol and acetonitrile. Lipids were detected by an Orbitrap mass spectrometer operated in MS mode, with a scan range (m/z) 200-1200, and a maximum injection time of 50 ms. The higher–energy collisional dissociation, which facilitated high collision energy ion fragmentation, was employed as a detector for MS/MS. The MS/MS scan range (m/z) was 50–1200 with a fragmentation energy of 25-40.

### Lipidomic data processing

The data were analyzed using LipidSearch software v 4.2 (Thermo, CA). Initially, automated processing of acquired mass spectra, retention time correction, peak alignment, and identification were carried out. MS/MS-based match was used for lipid identification. The mass tolerance was set at 5 ppm for both the precursor and the fragment. Lipid-dependent analysis was used to eliminate false positives.

### Atomistic models of membrane construction and system equilibration

The lipid bilayer composition was elucidated to consist of phosphatidylcholine (PC), phosphatidylethanolamine (PE), phosphatidylglycerol (PG), phosphatidylinositol (PI), and phosphatidylserine (PS), with varying quantities of each, in the membranes of neural and NSCs. For accurate simulation, the small-scale representative version of the membrane was modelled using Materials Science Suite, maintaining the lipid proportions. An appropriate number of lipids were randomly chosen from each class while maintaining the concentration gradients and diversity. The study aimed to elucidate the role of diverse membrane components in maintaining their innate stability and functionality upon interaction with another ligand.

Membranes were constructed using the specified lipid composition with the Structured Liquid Builder module. Ten thousand water molecules were added to each side of the membrane, ensuring the absence of undesired artifacts such as ring spears during system setup. The OPLS4 force field (38), as incorporated in Desmond (39), was used for parameterizing the system. The system was subsequently subjected to a multi-step relaxation protocol as outlined below:

Brownian Minimization (500 ps): The system underwent 500 ps of Brownian dynamics at 10 K; NVT Equilibration (2 ns): A relaxation step was performed in the NVT ensemble, maintaining a temperature of 100 K and utilizing a timestep of 1 fs; Gradual Heating in the NPT Ensemble: The system was progressively heated from 100 K to 300 K in three sequential steps under the NPT ensemble with a timestep of 1 fs and pressure of 1.013 bar; Equilibration (100 ns): The system was equilibrated for 100 ns in the NPT ensemble at 310 K and 1.013 bar, employing semi-isotropic pressure coupling and a 1 fs timestep.

### Identifying GXM interaction with membrane lipids

The equilibration of the membrane was verified by calculating the area per lipid, which was determined using the atomic coordinates of the lipid head groups. Once equilibration was confirmed, 1 GXM (Serotype D, 40) molecule was incorporated into the system using the Disordered System Builder module of the suite. A subsequent production run was carried out for 200 ns under NPT conditions at 310 K and 1.013 bar, maintaining semi-isotropic pressure coupling. All analyses were performed using the tools provided within the Materials Science Suite.

### Statistical analysis

GraphPad Prism software was used to analyze the data statistically. One-way ANOVA was used to compare data from three or more groups. An unpaired t-test was used to determine the significance between groups in studies involving cell proliferation, cell death, western blot, and lipidomics data. P values < 0.05 were considered significant.

## Results

### Capsular GXM does not alter NSC proliferation but induces cell death

**Figure 1:**
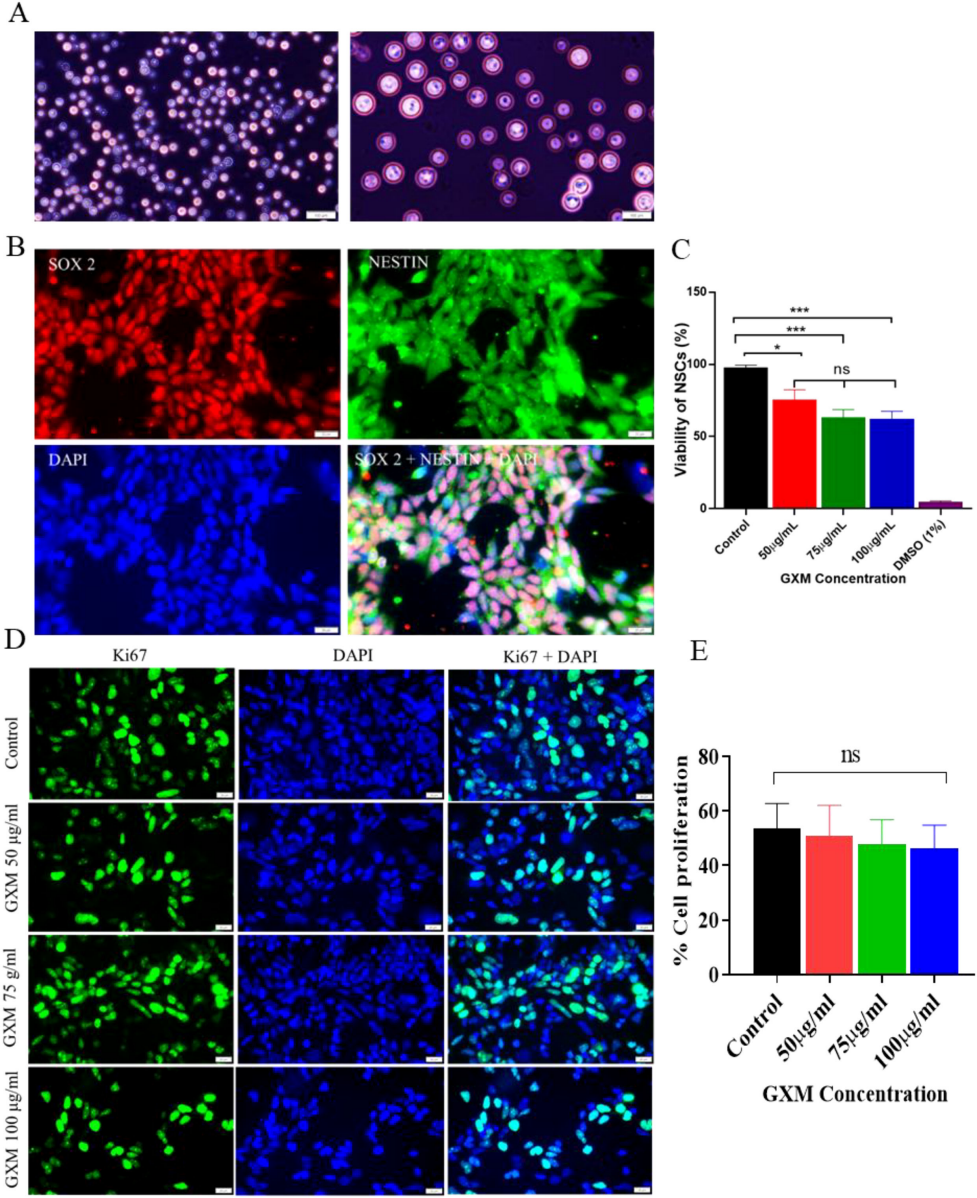
The impact of GXM isolated from *Cryptococcus neoformans* on the viability and proliferation of neural stem cells. **(A)***C. neoformans* (ATCC 32045) was grown in Yeast Peptone Dextrose medium, stained with mucicarmine to observe its capsular structure. **(B)** NSCs were generated from human-induced pluripotent stem cells; identity of the generated NSCs was confirmed by dual immunostaining with markers SOX2 (red), NESTIN (green), and DAPI (blue). The merged image demonstrates cells positive for SOX2, NESTIN, and DAPI (pink). **(C)** NSCs were exposed to different concentrations of GXM (50-100 μg/ml) for 48 hours and their viability was assessed by MTT assay, which was significantly reduced upon GXM treatment, compared to controls. However, a dose-dependent reduction in viability was not observed above 75 μg/ml of GXM, possibly due to its activity saturation. As a positive control, 1% DMSO was used. **(D)** To check the direct impact of GXM on NSCs proliferation, 2D cultured NSCs were treated with varying concentrations of GXM (50 μg/ml, 75 μg/ml, and 100 μg/ml) for 48 hours. The left panel images show Ki67-positive cells. The middle panel displays DAPI-positive cells, and the right panel shows merged images of both Ki67- and DAPI-positive cells. **(E)** To quantify the percentage of proliferation, the number of Ki67-positive cells was counted in relation to DAPI-positive cells, and the graph shows no significant reduction in the proliferation of NSCs at the studied doses. *p<0.05; ***p<0.001. ns= non significant.

*C. neoformans* were grown in the YPD medium (**Figure 1A**), from which GXM was extracted following the protocol described. Human neural stem cells (NSCs) were derived from induced pluripotent stem cells (iPSCs). The identity of cultured NSCs was confirmed through SOX2-NESTIN dual immunostaining (**Figure 1B**). Evaluation of the viability of NSCs at 48 h following GXM treatment (50-100 μg/ml) by MTT assay revealed a significant reduction in viability at 50 μg/ml of GXM compared to the control group (n=3/group). Additionally, increasing the dose of GXM to 75 μg/ml and 100 μg/ml demonstrated a nonsignificant reduction in viability. However, a dose-dependent reduction in viability was not observed between 75 μg/ml and 100 μg/ml of GXM (**Figure 1C**), possibly due to a saturation in the inhibitory activity of this polysaccharide. Further, NSCs were evaluated for the direct impact of varying doses of GXM on their proliferation. Cells were treated with 50, 75, and 100 μg/ml of GXM for 48 hours and then immunostained with Ki67, the cell proliferation marker (**Figure 1D**). Quantitation of Ki67-positive cells in DAPI counterstained slides identified no significant reduction in the proliferation of NSCs at the studied doses (**Figure 1E**). Among the assessed doses of GXM, since 50 μg/ml induced no alteration in NSC proliferation but significantly reduced their viability, this concentration of GXM was selected for further studies with organoids.

### Cell organoids lacking microglia were used to mimic microglial dysfunction

Earlier studies demonstrated that (i) microglia are nonprotective against *C. neoformans* infection, and (ii) GXM in the brain further enhances the *C. neoformans* invasion into the brain and causes defective immunity with microglial dysregulation. To understand the cellular pathogenesis of *C. neoformans* under microglia non-functional conditions, we utilized the bona fide protocol of Lancaster and Knoblich (30) to generate cerebral organoids, which lack cells of mesodermal origin due to the early embedding of embryoid bodies in matrigel. We used 60-day-old organoids, as they provide an ideal opportunity to study the interaction of GXM with abundant neurons, astroglia, and neural stem cells, without the presence of microglia.

**Figure 2:**
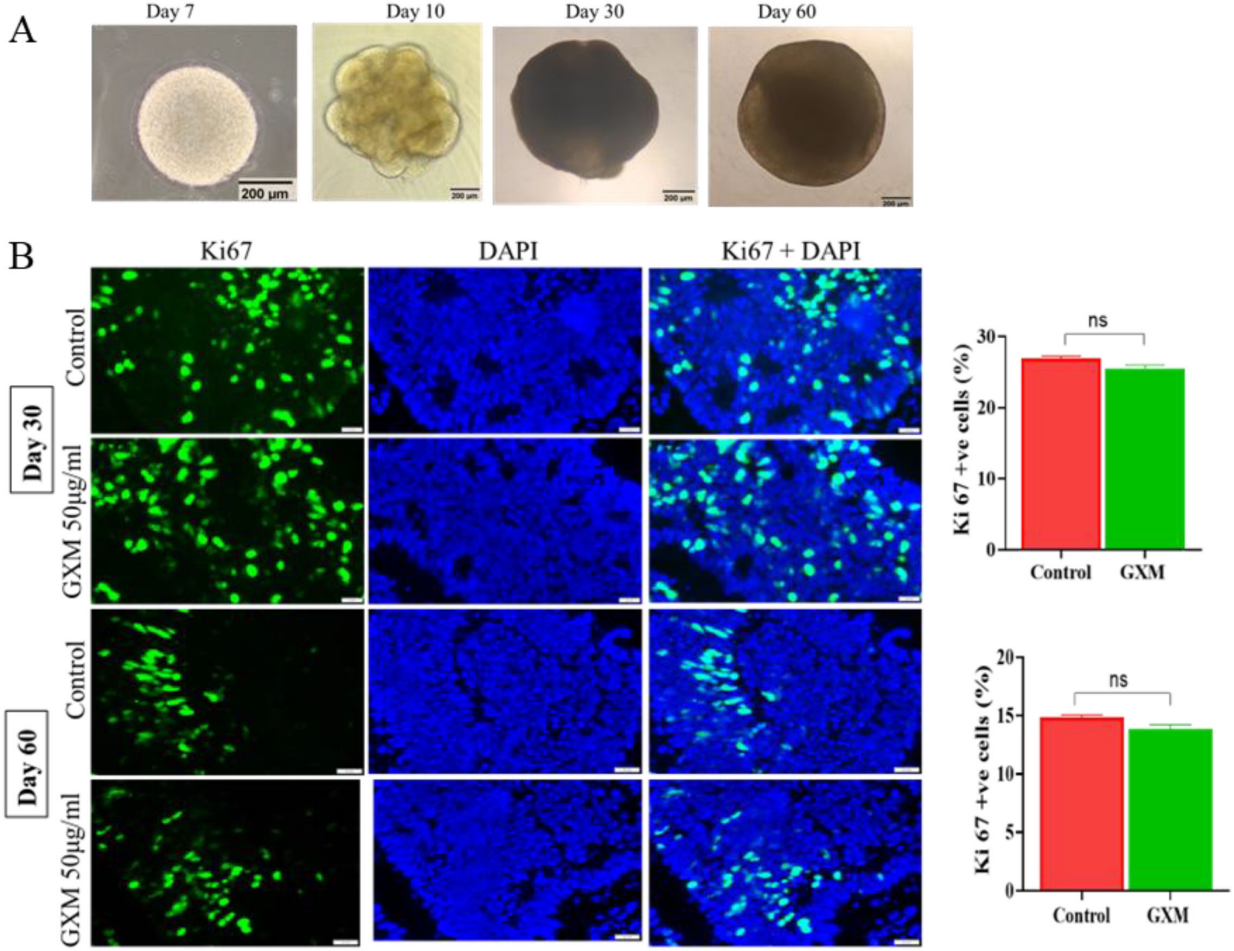
Impact of GXM on cell proliferation in cerebral organoids. **(A)** Generation of cerebral organoids from human-induced pluripotent stem cells. The Day 7 image shows an embryoid body generated from hiPSCs. The embryoid body was then embedded into a Matrigel droplet, and the day 10 image shows the growth of a neuroepithelial bud surrounding fluid-filled cavities. The cerebral organoids were grown for up to 2 months. **(B)** To evaluate the cell proliferation in cerebral organoids post-GXM exposure, day 30 and day 60 cerebral organoids were treated with 50μg/ml of GXM for 48 hours. Cryosections were taken and immunostained with Ki-67 (green) and DAPI (blue) in both the treated and untreated groups (n = 6/group). Merged images demonstrate cells positive for both Ki67 and DAPI. The right panel graph depicts the percentage of proliferation in the control and GXM-exposed group. The Ki-67-Positive cells were counted in relation to DAPI-positive cells in both groups. There was no significant difference in the proliferating cell population upon GXM treatment compared to controls, as observed in day 30 and day 60 organoids (n = 6 organoids/group/time point). ns= non significant.

Cerebral organoids were generated using iPSCs (**Figure 2A**), which displayed self-organizing internal structures with fluid-filled cavities. They were characterized for the presence of neural stem cells, neurons, and glia at days 30 and 60. On days 30 and 60, organoids were largely positive for Tuj1. Radial glial cells surrounding the fluid-filled cavities were positive for SOX2. The extensive presence of GFAP-positive astrocytes was seen in the day-60 organoids. Day-60 organoids also demonstrated the presence of Tuj1-positive cells interspersed with GFAP-positive cells. Additionally, many Ki-67+ proliferating cells remained within the organoids, along with Tuj1-positive neurons, at day 60. Markers positive for microglial cells (IBA1) and oligodendrocytes (SOX10) were undetected in day 30 and day 60 organoids, and the data are not presented here.

Following treatment with GXM, to track its localization inside the cerebral organoids, a monoclonal antibody was used to label GXM. The Alexa Fluor 488-labelled GXM was detected in both superficial layers and deep within the organoids, as identified through green fluorescence (**Supplementary Figure S1**). First, to evaluate the direct impact of GXM on proliferating neural stem cells within cerebral organoids, day-30 and day-60 organoids were exposed to 50 μg/ml of GXM for 48 hours. Following fixation and cryosectioning, the organoids were immunostained for Ki-67, and the number of positive cells was counted in relation to DAPI-positive cells. The percentage of proliferation was then assessed. Similar to the results of the 2D culture, no significant difference in proliferating cell population was observed upon GXM treatment (50µg/ml) compared to the control group on day-30 and day-60 organoids (n=6 organoids/group/time point) (**Figure 2B**). Fluorescence images and data analysis from 2D cultures and the cerebral organoids confirmed the minimal impact of GXM on neural stem cell proliferation.

**Figure 3:**
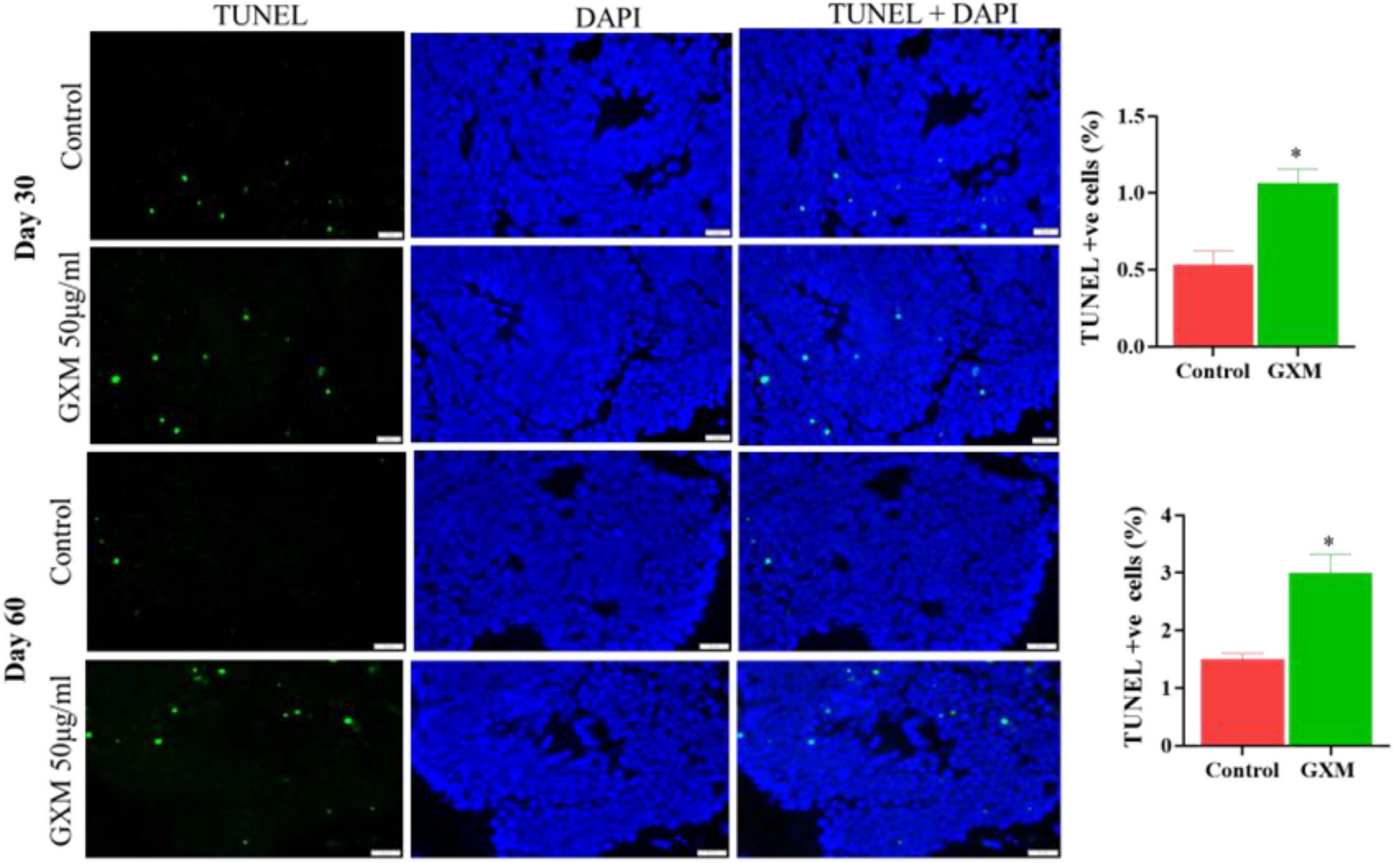
Impact of GXM on cell death in cerebral organoids. To identify the cell death in cerebral organoids post-GXM exposure, day 30 and day 60 cerebral organoids were exposed to 50μg/ml of GXM for 48 hours, and cell death was analyzed using the TUNEL assay in both the control and GXM exposure groups. Fluorescent images represent TUNEL-positive (green) cells and DAPI-positive nuclei (blue), and the merged image illustrates TUNEL- and DAPI-co-stained positive cells. The right panel graph depicts the percentage of cell death at both time points. There was a moderate but significant increase in cell death upon GXM treatment compared to controls on days 30 and 60, as observed in cerebral organoids (n = 6 organoids/group/time point). *p<0.05.

As GXM reduced cell viability in 2D cultures, to identify the direct impact of GXM in the cerebral organoids, day-30 and day-60 organoids were exposed to 50μg/ml of GXM for 48 hours. Following fixation and cryo-sectioning, a TUNEL assay was performed in the organoids to detect the amount of GXM-induced cell death (**Figure 3**). Green fluorescent images represent TUNEL-positive cells and blue fluorescent images represent DAPI-positive nuclei while the merged image illustrates co-labeling of TUNEL and DAPI. Quantitation of TUNEL-positive cells against DAPI-positive cells indicated a moderate but significant increase in cell death upon GXM treatment (50µg/ml) compared to controls on day-30 and day-60 cerebral organoids (n=6 organoids/group/time point) (**Figure 3**). Results from 2D NSC culture viability studies and TUNEL assay studies with cerebral organoids at days 30 and day-60 confirmed that GXM induces cell death.

### GXM does not target proliferating cells or astrocytes in cerebral organoids

**Figure 4:**
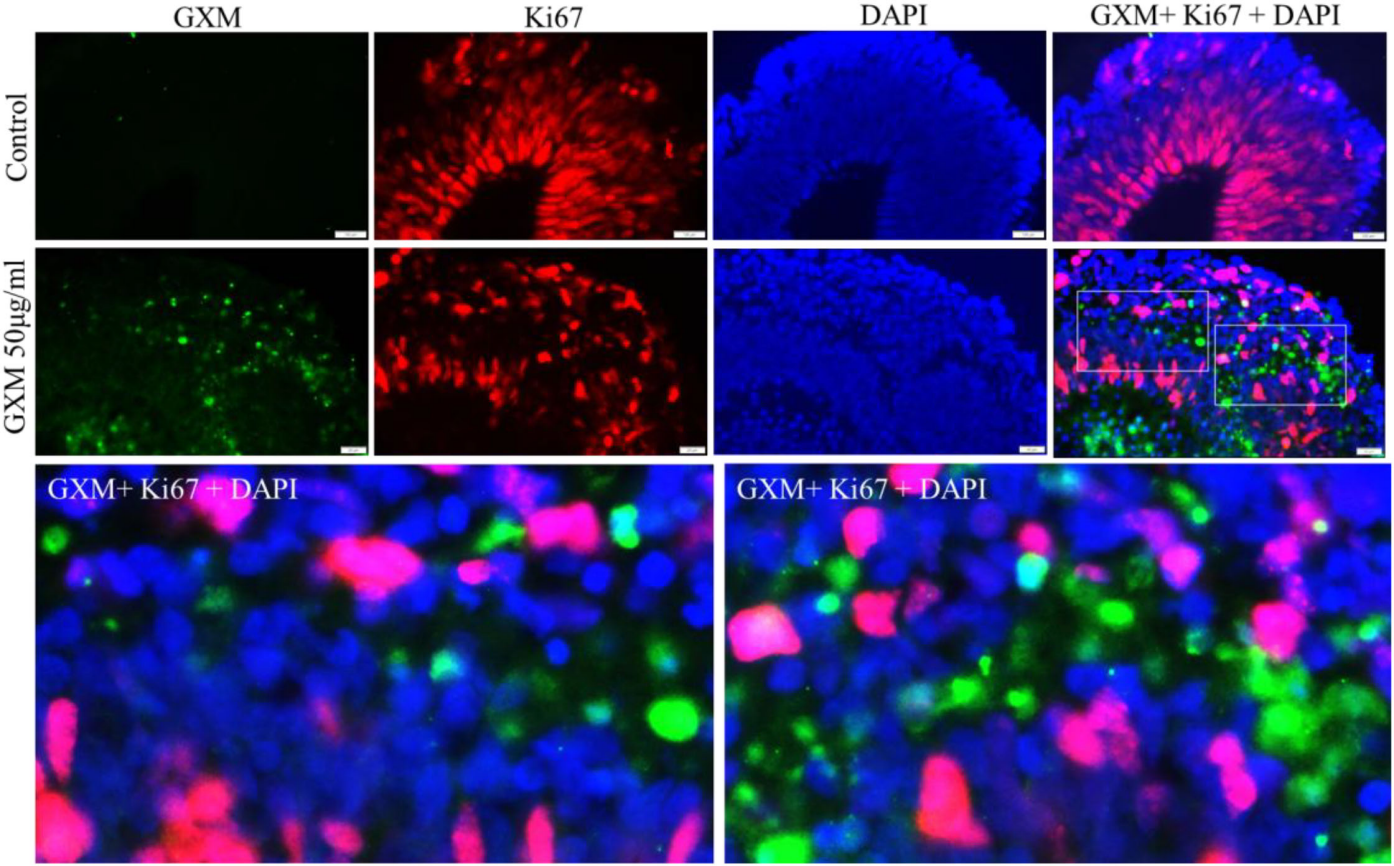
Localization of GXM with different cell types in cerebral organoids. Day 60 cerebral organoids were exposed to 50 µg/ml GXM for 48 hours. Dual immunostaining was performed using Ki67 (red) and an anti-GXM antibody (green) in both treated and untreated cerebral organoids (n = 6/group). The merged image demonstrates no or minimal co-localization of Ki67 (red) with GXM (green). The lower panel represents the selected fields from the merged image, highlighting the GXM and proliferating cells.

Following treatment with GXM, the study tried to identify the targets of GXM among different cell types in the cerebral organoids. To understand cell types prone to GXM, day-60 cerebral organoids were exposed to 50 µg/ml GXM, for 48 hours. GXM treated and untreated cerebral organoids (n=6/group) were immunostained with anti GXM antibody along with Ki-67, that labels the proliferating cells in the organoids. Green fluorescent labelled GXM was identified throughout the day-60 cerebral organoid **Figure 4**). Red fluorescent labelled Ki-67 positive cells were seen abundantly in the organoids (**Figure 4**). Merged images represent co-labeling of GXM with Ki-67. Although GXM was observed within the vicinity of Ki67-positive cells, no or minimal colocalization of Ki67 with GXM was observed in all the organoids (**Figure 4**). This indicated that GXM do not target the proliferating cells in the cerebral organoids.

**Figure 5:**
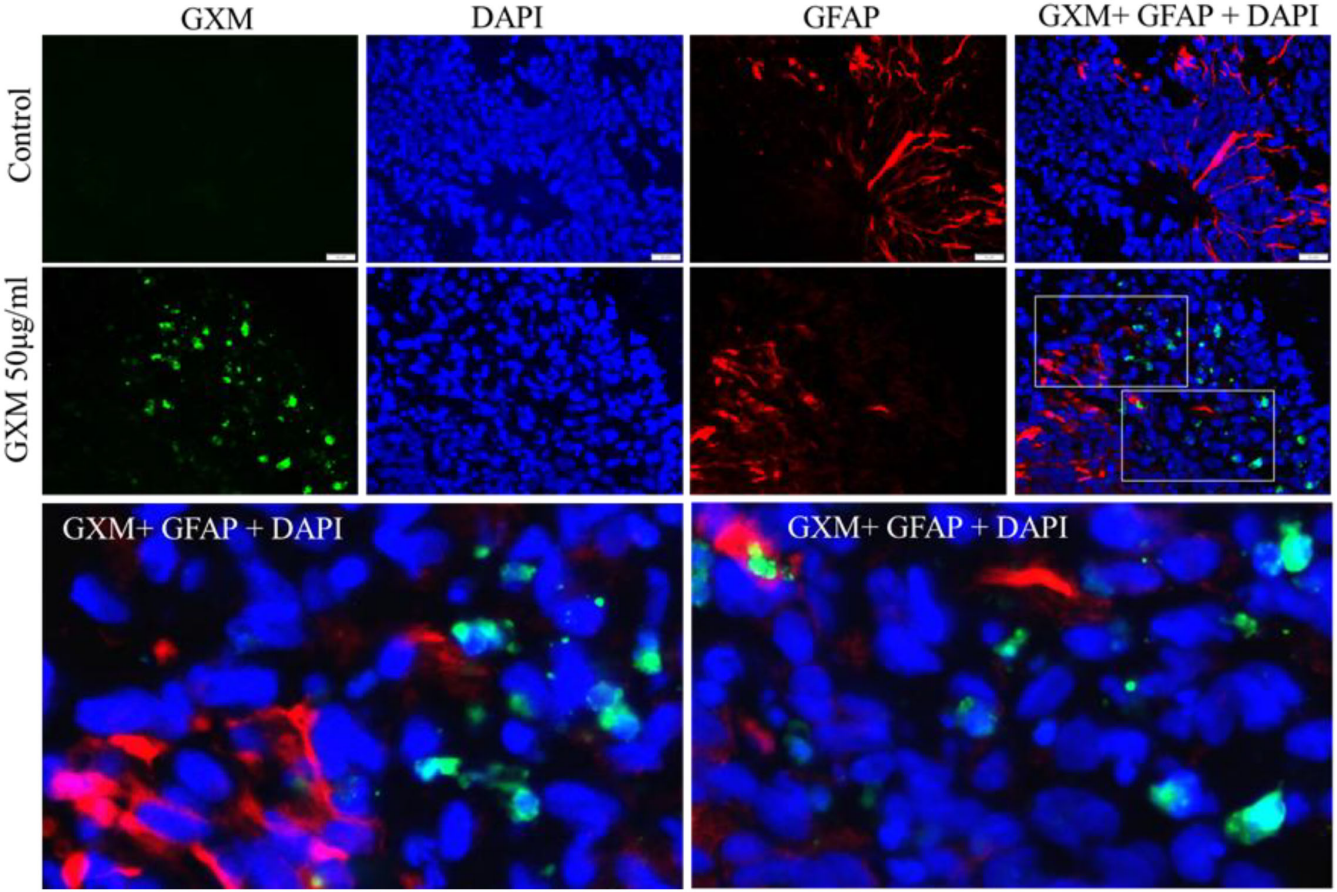
Localization of GXM with different cell types in cerebral organoids. Day 60 cerebral organoids were exposed to 50 µg/ml GXM for 48 hours. Dual immunostaining was performed using an antibody for astrocytes (GFAP, red), an anti-GXM antibody (green), and DAPI (blue) in both the treated and untreated groups (n = 6/group). The merged image shows no co-localization of GFAP (red) with GXM (green). The lower panel represents the selected fields from the merged image, highlighting that the GXM has not co-localized with GFAP-positive astrocytes.

GXM treated (50 µg/ml) and untreated cerebral organoids (n=6/group) were immunostained with anti GXM antibody along with antibody for the astrocyte marker GFAP. Green fluorescent labelled GXM was identified throughout the day-60 cerebral organoid (**Figure 5**). Red fluorescent labelled GFAP positive cells were seen abundantly in the organoids (**Figure 5**). Merged images represent co-labeling of GXM with GFAP. Again, GXM was observed within the locality of GFAP positive cells. However, minimal colocalization of GFAP with GXM was observed in all the organoids (**Figure 5**). This indicated that GXM do not target the astrocytes in the cerebral organoids.

### GXM showed affinity toward neurons in 3D cerebral organoids

**Figure 6:**
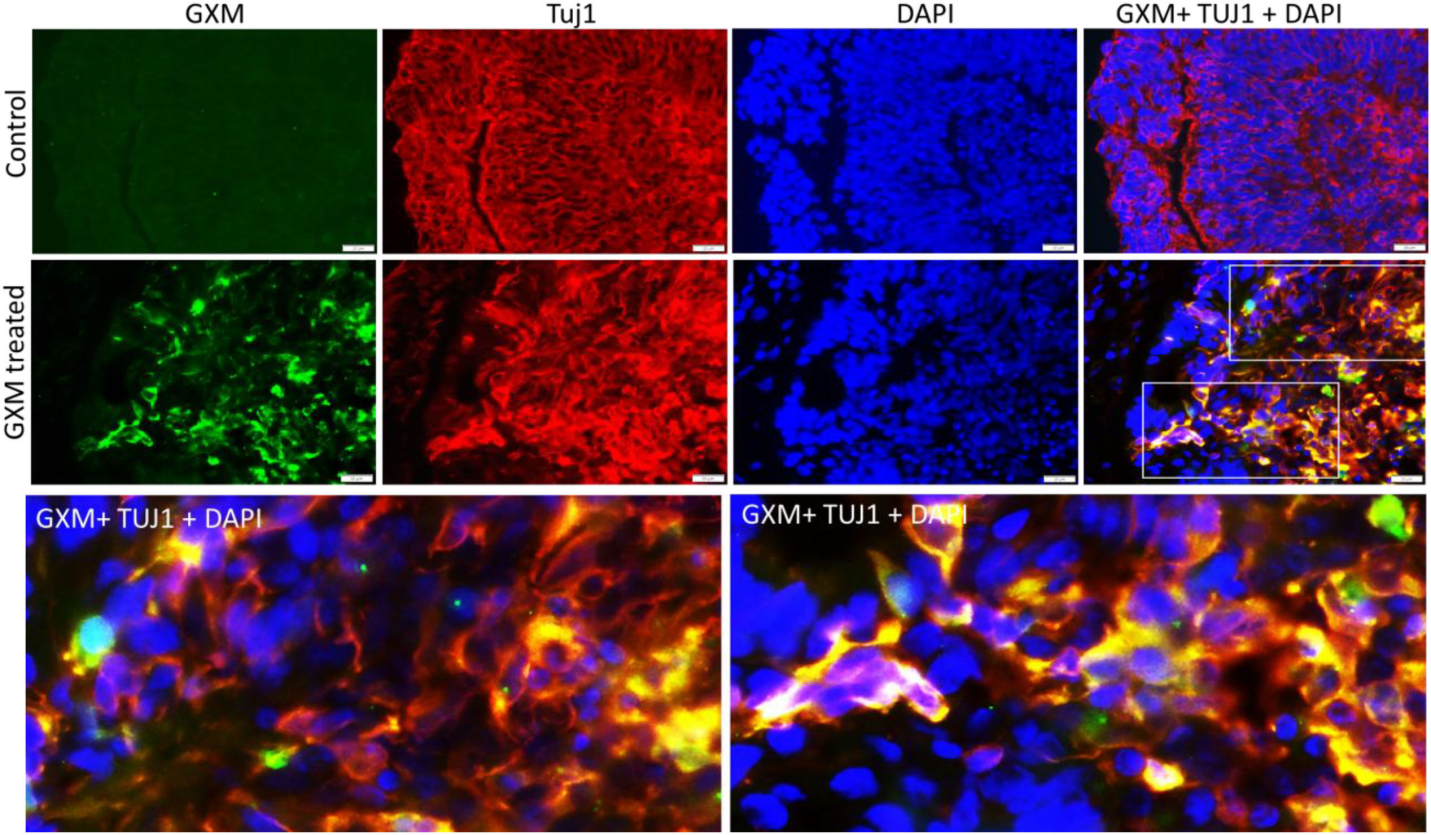
Co-localization of GXM with neurons. Dual immunostaining was performed on day 60 cerebral organoids with TUJ1 antibody (red) for neurons with anti-GXM antibody (green) at 48 hours following GXM exposure (50µg/ml) and compared with controls (n=6/group). Merged images demonstrate the co-localization of GXM with TUJ1-positive cells (orange). Lower panels show selected fields highlighting the association of GXM with neurons.

Since GXM didn’t show much affinity to proliferating cells and astrocytes, we further tested its impact on neurons. Day 60 cerebral organoids were dual immunostained with anti GXM antibody along with antibody for the neuronal marker Tuj1 at 48 hours of GXM treatment (50 µg/ml) and compared with controls (n=6/group). Green fluorescent labelled GXM was identified throughout the day-60 cerebral organoid (**Figure 6**). Red fluorescent labelled Tuj1 positive cells were seen abundantly in the organoids (**Figure 6**). Tuj1 colocalized with GXM in several places (**Figure 6**) but was primarily seen on the membranes and not inside the cytoplasm or nucleus (**Figure 6**, inserts). While GXM is primarily targeted to neurons, not all neurons were targeted by GXM. Also, GXM is sometimes associated with other cell types, such as astrocytes and proliferating cells. Together, these data demonstrate that GXM preferentially targets neurons within organoids, while neural stem cells or astrocytes are minimally targeted (**Figure 6**).

### GXM affects synaptic vesicle protein levels in neurons

**Figure 7:**
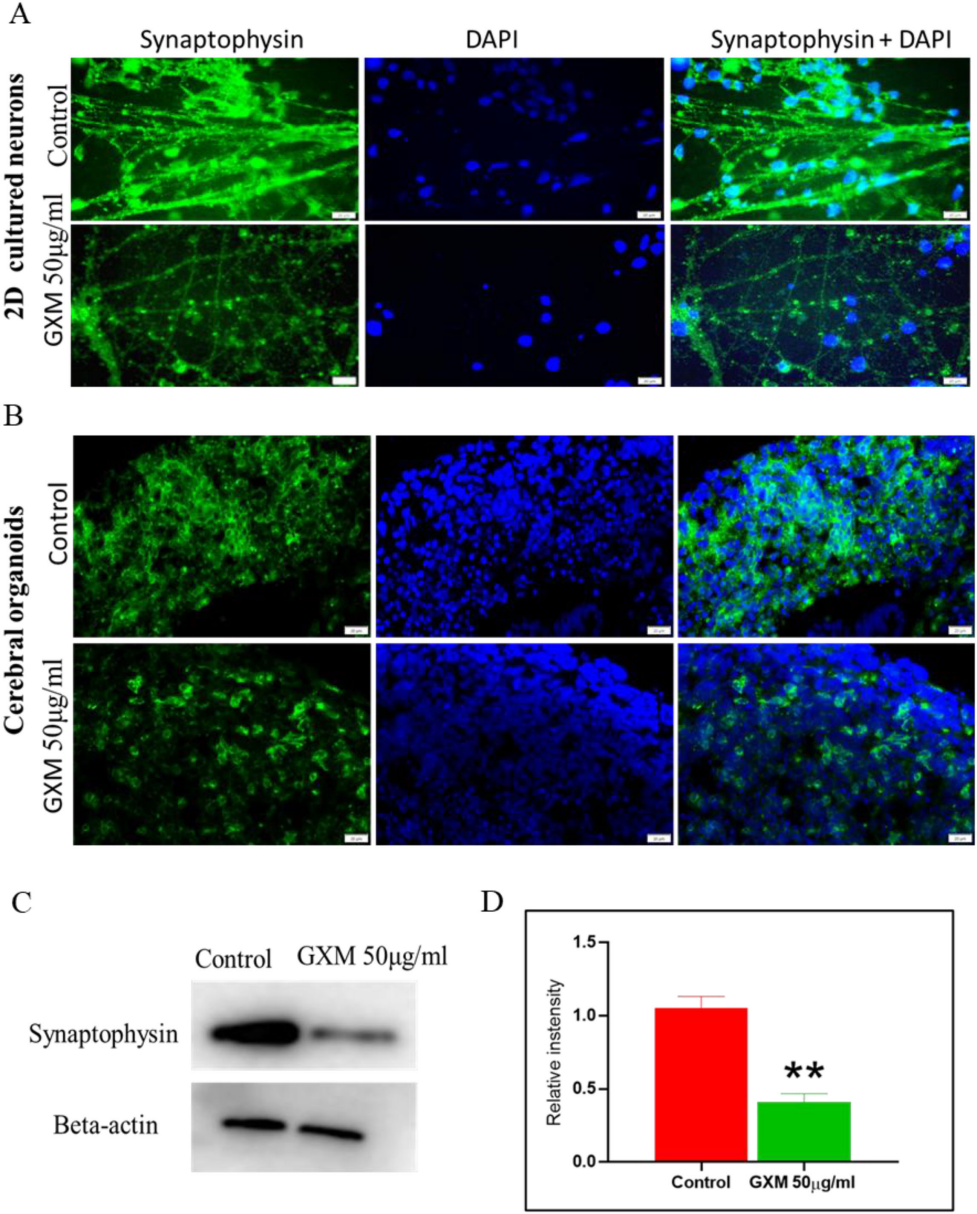
GXM affects the levels of synaptic vesicle proteins in neurons. **(A)** The level of synaptophysin was assessed on day 21 neurons generated from induced pluripotent stem cells. Neurons were exposed to 50 µg/ml GXM for 48 hours compared with controls. Fluorescent images showed synaptophysin (1:200) (green) positive neurons and DAPI (blue). Merged images demonstrate both synaptophysin and DAPI-positive cells. **(B)** Fluorescent images of synaptophysin in cerebral organoids post-GXM exposure. The upper panel shows the control group, and the lower panel represents day 60 cerebral organoids exposed to 50 µg/ml GXM for 48 hours. The merged image demonstrates both synaptophysin and DAPI. **(C)** The neuronal level of synaptophysin was quantified using western blot after 48 hours of GXM (50µg/ml) exposure. **(D)** The values in the bar charts, represented as mean ± S.E.M for triplicate experiments, show a significant reduction in the level of synaptophysin in the GXM exposure group compared to the control group (p = 0.0086). **p<0.01.

Mouse models of cryptococcal meningoencephalitis demonstrated severe synaptic deficits (41). Since GXM is the major virulent factor, this study assessed whether GXM targeting to neurons leads to neuronal dysfunction. Synaptophysin was considered, which is abundantly present in the integral membrane of the neuronal synaptic vesicles required for neuronal activity. Neurons were cultured for 21 days in a 12-well dish. GXM (50 µg/ml) was added to the cell culture media and incubated for 48 hours. Immunostaining with synaptophysin antibody demonstrated green fluorescence for synaptophysin and blue fluorescence for DAPI. In the neurons treated with GXM, visibly reduced synaptophysin levels were observed compared to controls (**Figure 7A**). To confirm that GXM interaction reduces synaptophysin, synaptophysin levels were measured in the day-60 cerebral organoids treated with GXM. Similar to 2D neurons, a reduced synaptophysin expression was observed in the organoids treated with GXM compared to untreated neurons (**Figure 7B**). This data was further assessed by analyzing the level of synaptophysin using western blotting in 2D cultured neurons (**Figure 7C**) and its quantification using Image J, which confirmed that synaptophysin levels are significantly reduced (**Figure 7D**, p=0.0086) following GXM exposure. Overall, these results demonstrates that GXM treatment reduces synaptophysin levels in neurons.

### Reversal of the key components between NSCs and neurons and lipid selection for membrane building

**Figure 8:**
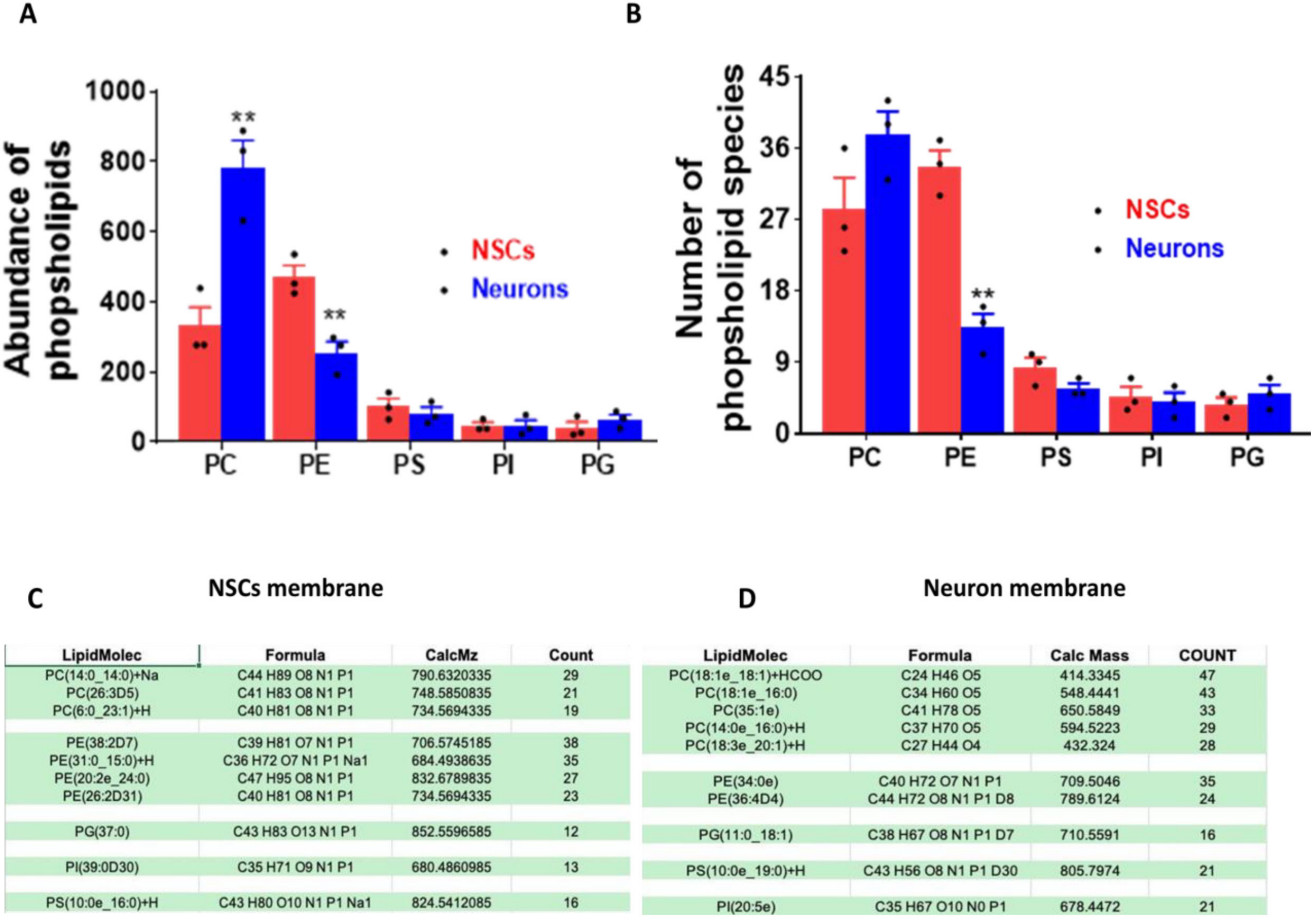
Untargeted lipidomics of NSCs and neurons. **(A)** Untargeted lipidomics of NSCs and neurons identified a 2.4-fold higher abundance of phosphatidylcholine (PC) in neurons than in NSCs. At the same time, phosphatidylethanolamine (PE) levels were ~1.8-fold lower in neurons compared to NSCs. The abundance of phosphatidylglycerol (PG), phosphatidylinositol (PI), and phosphatidylserine (PS) remains unchanged between the groups. **(B)** Apart from total abundance, the number of PC species is also higher, and PE species are lower in neurons than in NSCs. The number of PG, PI, and PS remains unchanged between the groups **p<0.01. **(C)** Neuronal and **(D)** NSCs membrane composition used for membrane construction based on lipids from each class while maintaining the proportional concentration and diversity, based on the untargeted lipidomics.

As consistent cell receptors for GXM are not detected, to identify the rationale of GXM affinity towards neurons rather than other cell types, we considered the membrane composition of neural stem cells and neurons as key players. Untargeted lipidomics was performed for iPSC-derived neural stem cells and neurons. The study analyzed neutral lipids and phospholipids, as neutral lipids are generally present in intracellular compartments as lipid droplets (42, 43), whereas phospholipids are the major constituents of membranes. The difference between the two groups identified was a ~2.4-fold higher abundance of phosphatidylcholine (PC) in neurons than NSCs (p<0.01), whereas phosphatidylethanolamine (PE) levels were ~1.8-fold lower in neurons compared to NSCs (p<0.01) (**Figure 8A**). Apart from the total abundance, PC was higher, and PE was lower in neurons than in NSCs (**Figure 8B**). Further analysis revealed that among the PCs, neurons have a significantly higher number of very long-chain fatty acids compared to NSCs (p<0.05). In contrast, the levels of medium- and long-chain fatty acids remained unchanged between groups. Moreover, among the PEs, medium-chain fatty acids were significantly lower in neurons than in NSCs (p<0.05). The abundance and fatty-acid chain lengths of phosphatidylglycerol (PG), phosphatidylinositol (PI), and phosphatidylserine (PS) remained unchanged between neurons and NSCs (**Figures 8A, B**).

To identify the impact of lipid composition on GXM interaction, the study planned to build a lipid membranes based on proportional lipid compositions determined through untargeted lipidomics data. The lipid composition elucidated consists of PCs, PEs, PGs, PIs, and PS, varying quantities of each, in human neurons and NSCs (**Figures 8C, D**).

### First-ever atomistic models of NSC and neuronal membranes demonstrate the strong binding energy of GXM with phosphatidylcholine-rich neuronal membranes

In a novel approach, this study presents the first-ever atomistic models of neuronal and NSC membranes and their interaction with GXM. Both the neuron and NSC membrane models were built using the Structured Liquid Builder (44) in the Materials Science Suite (45) based on the original untargeted lipidomics data. This model preserved the proportional number of lipids from each class while maintaining the concentration gradients and diversity as discussed earlier.

**Figure 9:**
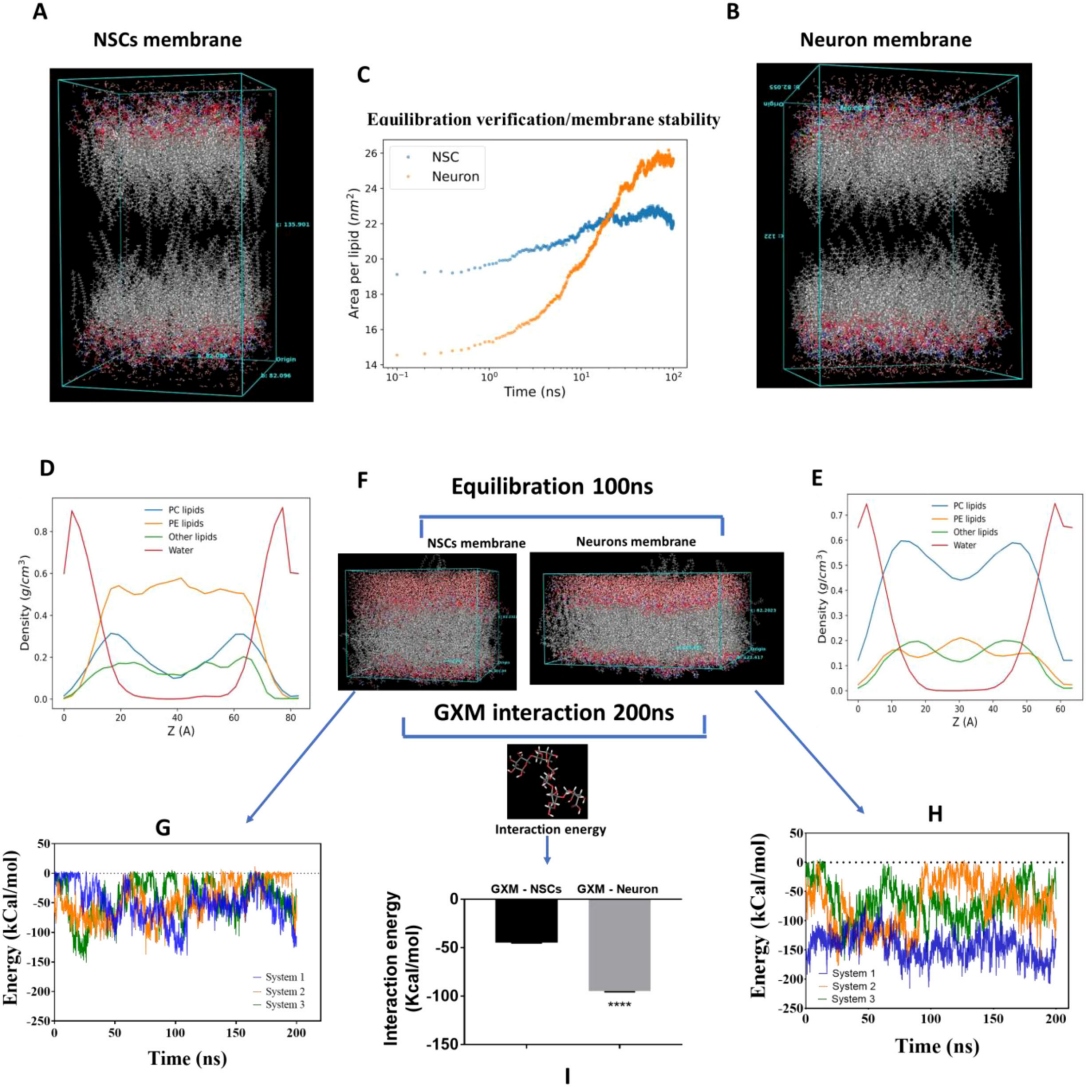
Construction of neuronal and neural stem cells membranes and their interaction with GXM. **(A, B)** Constructed NSC and neuronal membranes, respectively. **(C)** Area per lipid (APL) was determined as a function of the simulation time using the atomic coordinates of the lipid head groups. **(D, E)** Density profile for NSCs and neuronal membranes to verify equilibration of the membranes. **(F)** A single molecule of GXM was incorporated into the equilibrated membrane system using the Disordered System Builder in the MS Maestro suite. Subsequently, a production run of 200 ns with both membranes was performed for further analysis. **(G)** Interaction energy of GXM with NSCs. **(H)** Interaction energy of GXM with neurons. **(I)** A comparison of interaction energy between the neuronal membrane and GXM versus NSCs identified a strong attractive interaction energy for GXM with the neuronal membrane compared to NSCs. ****p<0.0001.

Both membranes (**Figures 9A, B**) were subjected to a similar equilibration protocol (see Methods). Equilibration of the membranes was confirmed by monitoring the area per lipid (APL), calculated using the lipid head groups. The neuronal membrane exhibited a higher APL compared to the NSC membrane, indicating that NSC membranes are more densely packed (**Figure 9C**). This is possibly due to the presence of more unsaturated and very long-chain fatty acids in neurons than in NSCs (p < 0.05). At equilibrium, the density profile showed that PC and PE had higher densities than PG, PS, and PI across both membrane types, with an inverse relationship to water, suggesting a hydrophobic core (**Figures 9D, E**). The neuron membrane possessed a higher quantity of PC than the rest, as depicted by twin peaks in the graph. In comparison, the NSC membrane possessed PE in greater quantity (**Figures 9D, E**).

A single molecule of GXM was incorporated above the membrane surface using the Disordered System Builder module of the suite following equilibration (**Figure 9F**). Three different initial configurations for GXM relative to the membrane were chosen for each system. Subsequently, a production run of 200 ns with both membranes was performed for further analysis. **Figures 9G, H** present the interaction energy (van der Waals and electrostatic) for each system across three replicates as a function of simulation time. Observed variation in the evolution of interaction energies among different configurations highlights a degree of configuration dependence. However, averaging across replicates mitigates these differences, providing a more robust representation of the system behavior. A comparison identified a strong attractive interaction energy (p<0.0001) for GXM with neuronal membrane compared to NSCs (**Figure 9I**). This identifies that lipid membrane components decide the GXM interaction. Furthermore, the interaction energy of GXM with PC alone accounted for approximately 30-35% of the total interaction energy, indicating that higher PC levels are primarily responsible for GXM interaction and its higher affinity to neuronal membranes.

## Discussion

GXM is a high molecular mass capsular polysaccharide and a major virulent factor of *C. neoformans.* Its presence in the brain is reported to enhance the *C. neoformans* invasion into the brain and cause defective immunity, accompanied by microglial dysregulation (13, 21). However, microglia have been shown to migrate away from GXM accumulation sites (13), and they are unable to remove accumulated GXM. In this study, using a cerebral organoid model devoid of microglia and 2D-cultured NSCs and neurons, we demonstrate that GXM exposure induces subtle cell death, but progenitor cell proliferation remains unaffected. Interestingly, GXM preferentially targets neurons irrespective of the presence/abundance of NSCs and astrocytes. Synaptophysin, an integral component of neuronal synaptic vesicles, was significantly reduced upon GXM exposure. Furthermore, a comparative membrane composition analysis by untargeted lipidomics revealed higher phosphatidylcholine and lower phosphatidylethanolamine levels in neurons compared to NSCs. The study utilized the first-ever atomistic models of neuronal and NSC membranes built using a proportion of the original lipid compositions, to identify the interactions between GXM and the membranes using the Materials Science Suite. GXM exhibited a strong attractive interaction energy with neuronal membranes, with phosphatidylcholine identified as the primary contributor to this interaction. This study provides primary evidence that lipid membranes containing higher phosphatidylcholine are a novel target of GXM in the pathogenesis of *C. neoformans.* Together, this study identifies two essential phenomena during *C. neoformans* pathogenesis: (i) specific targeting of GXM that affects synaptic deficit in neurons, a major reason for the neurological dysfunctions visible in *C. neoformans-induced* meningoencephalitis, and (ii) primary evidence of lipid membrane rich in phosphatidylcholine as a novel target of GXM for pathogenesis.

The impact of GXM on the survival and proliferation of target cells depends on multiple factors. For example, following infection, GXM accumulates in several tissues for a long time and is cleared by resident macrophages, which also act as its reservoir, leading to their apoptosis through caspase-dependent and independent pathways (22, 46–49). Additionally, GXM is known to alter the physiological state of target cells, such as lung epithelial cells (50). GXM can be internalized by macrophages (51), and suppresses the host cell proliferation in a mechanism that includes apoptosis (49). Also, distinct cells behave differently to GXM exposure. For example, monocytes were utilized as reservoirs of GXM, but GXM can be quickly ingested, degraded, or expelled by neutrophils (52). While capsule components are used to evade recognition by hosts (53) they are also among the first components to interact with the host immune system (54). Capsular GXM is recognized by TLR2, CD14, and TLR-associated adaptor protein MyD88 (55, 56). *In vitro* cell line studies demonstrated an interaction between *C. neoformans* and astrocytes (57), while immunocompetent rats have shown glia-mediated inflammation (58). However, this capsular polysaccharide shows minimal interaction with astrocytes in postmortem brain samples of cryptococcal meningoencephalitis (16). While the interaction of GXM was primarily with neurons, a minimal but significant apoptosis was observed in the organoids exposed to GXM, while proliferating cells remained unaffected, likely due to dose and interaction-related reasons.

Neurocognitive impairments are well associated with CNS infections. Patients with cryptococcal meningitis have been known to show neurocognitive impairment and neuropsychological deficits depending on the severity of infection, despite the complete eradication of fungal cells from the brain with a standard cryptococcal treatment regimen (59–63). Since earlier mouse models demonstrated severe synaptic deficits during cryptococcal meningoencephalitis (41), the expression of synaptophysin, a major synaptic vesicle protein in neurons, was evaluated. Interestingly, post-GXM exposure showed a marked reduction in the protein in neurons, indicating that GXM alone can cause neuronal dysfunction. Synaptophysin is a calcium-binding glycoprotein, an integral membrane component of the neuronal synaptic vesicles, and a modulator of endocytosis. This is an important finding; synaptophysin is critically required for neurotransmission and cognitive functions and its levels can be improved by various nonpharmacological (64, 65) and pharmacological interventions (66–69), if required for preventing *C. neoformans* pathogenesis. The targeting synaptophysin approach requires significant attention and studies in this aspect.

As a major membrane component, lipids play a crucial role in the interaction between pathogens (70). Previous studies demonstrated that immunocompromised hosts with low CD4 lymphocyte counts show higher phosphatidylcholine in their subcortical brain regions (containing neuronal cell bodies and dendrites) and are highly penetrated by *C. neoformans* (71–73). Furthermore, based on the observation that GXM specifically targets neuronal membranes, it was hypothesized that identifying lipid composition in different cell types could give an idea of the impact of membrane composition on GXM affinity to neurons. Earlier reports in mice have identified relatively elevated phosphatidylcholine levels and reduced phosphatidylethanolamine levels in neurons compared to astrocytes, microglia, and oligodendrocytes (74). Also, phosphatidylcholine level was higher in rat neurons while phosphatidylethanolamine level was lower than in astrocytes (75). Since the culture contained proliferating cells that GXM does not target, untargeted lipidomics analysis of neural stem cells and neurons was performed to identify the possible impact of lipids, rather than repeating lipid analysis in astrocytes. The study revealed a significant reversal in the abundance of phosphatidylcholine and phosphatidylethanolamines between neural stem cells and neurons, while most other lipids remained unchanged. This suggests that higher levels of phosphatidylcholine in the neuronal membrane could be a possible reason for GXM targeting neurons. It was demonstrated that, in neurons, local synthesis produces half of the phosphatidylcholine that accumulates in distal axons. The other half is transported from neuronal soma and proximal axons (76). While the fat-binding capacity of polysaccharides, including phosphatidylcholine, is well established, earlier studies demonstrated phospholipids as a trigger for *C. neoformans* exopolysaccharide production and present as a danger signal for this fungal infection that triggers capsular enlargement to prevent entry (77). In this study, a comparison of the direct interaction between the neuronal membrane and GXM versus NSCs and GXM identified a strong interaction energy for GXM with the neuronal membrane, compared to NSCs, suggesting that lipid membrane components may determine the GXM interaction. The present data demonstrate that GXM of *C. neoformans* interacts strongly with neuronal membranes enriched with phosphatidylcholine. However, in meningoencephalitis, whether GXM targeting of phosphatidylcholine is used as a protective measure for the survival and propagation of the fungus requires further study. Additionally, further studies in animal models of *C. neoformans* infection are required to identify the fundamental relationship between the abundance of phosphatidylcholine, reduced levels of synaptophysin and cognitive dysfunction. As neuronal membranes of the brain can directly integrate dietary fatty acids transported through fatty acid transporters, further studies on phosphatidylcholine enhancements/depletion, and direct interactions at different degrees may identify therapeutic opportunities with phosphatidylcholine-mediated inhibition of the pathogenesis of *C. neoformans*.

Overall, this study provides novel evidence that lipid membranes containing higher levels of phosphatidylcholine are a primary target of GXM from *C. neoformans*, which could be the possible reason for the preferential targeting of GXM to neurons. Additionally, GXM induced synaptic deficits in neurons, which could be a significant factor contributing to the neurological dysfunctions observed in this fungal infection. One limitation of the study is that it used organoids without microglia, rather than using dysfunctional microglia. Animal models of *C. neoformans* infections have demonstrated dysfunctional microglia in the brain; however, microglia are not lacking. Since it is not easy to demonstrate an organoid model with reduced microglia, we considered using organoids without microglia as a model to represent a condition of dysfunctional microglia. Further, the present study used the ATCC 32045 C*. neoformans* strain, as it is a well-characterized reference strain, widely used in studies investigating virulence factors and host-pathogen interactions in Cryptococcus species. The hybrid nature of this strain presents an opportunity to study strain variability and its impact on neuronal cytotoxicity—an aspect that has not been extensively reported in previous studies. The structure and physiological properties of GXM exhibit remarkable heterogeneity. There are considerable variations between serotypes and among strains within a single serotype. These differences can affect its interactions within the host environment (17, 78–80). The serotypes A/D are relatively less common compared to pure serotype A but are still clinically relevant (81, 82). Therefore, the strain used can be considered a strain seen in both clinical and environmental settings. However, the fungal strain may influence the extent of observed neuronal cell death. Comparative studies involving serotype A (H99), and clinical isolates could identify the strain-specific differences in GXM-mediated neuronal response. Also, GXM from the multiple strains of Cryptococcus *neoformans* and Cryptococcus *gatti*, can be considered to better elucidate species-specific differences in the outcome.

## Data Availability

The original contributions presented in the study are included in the article/**Supplementary Material**. Further inquiries can be directed to the corresponding author.

## References

[1] Hurtado JC Castillo P Fernandes F Navarro M Lovane L Casas I . Mortality due to Cryptococcus neoformans and Cryptococcus gattii in low-income settings: an autopsy study. Sci Rep. (2019) 9:7493. doi: 10.1038/s41598-019-43941-w, PMID: 31097746 PMC6522501

[2] Siqueira LPM Gimenes VMF de Freitas RS Melhem MSC Bonfietti LX da Silva AR . Evaluation of Vitek MS for Differentiation of *Cryptococcus neoformans* and *Cryptococcus gattii* Genotypes. J Clin Microbiol. (2019) 57:e01282–18. doi: 10.1128/JCM.01282-18, PMID: 30429250 PMC6322467

[3] Yu CH Sephton-Clark P Tenor JL Toffaletti DL Giamberardino C Haverkamp M . Gene expression of diverse cryptococcus isolates during infection of the human central nervous system. mBio. (2021) 12:e0231321. doi: 10.1128/mBio.02313-21, PMID: 34724829 PMC8561399

[4] Coussement J Heath CH Roberts MB Lane RJ Spelman T Smibert OC . Current epidemiology and clinical features of cryptococcus infection in patients without human immunodeficiency virus: A multicenter study in 46 hospitals in Australia and New Zealand. Clin Infect Dis: an Off Publ Infect Dis Soc America. (2023) 77:976–86. doi: 10.1093/cid/ciad321, PMID: 37235212

[5] Venkatareddy MP Upadhya D Yegneswaran PP Varghese A Pahadasingh S Prabhu AN . Molecular diagnostic methods for rapid diagnosis of central nervous system infections. Front Med Technol. (2025) 7:1497512. doi: 10.3389/fmedt.2025.1497512, PMID: 40400662 PMC12093496

[6] World Health Organization . WHO fungal priority pathogens list to guide research, development and public health action (2022). WHO. Available online at: https://www.who.int/publications/i/item/9789240060241 (Accessed July 10, 2025).

[7] Rajasingham R Govender NP Jordan A Loyse A Shroufi A Denning DW . The global burden of HIV-associated cryptococcal infection in adults in 2020: a modelling analysis. Lancet Infect Dis. (2022) 22:1748–55. doi: 10.1016/S1473-3099(22)00499-6, PMID: 36049486 PMC9701154

[8] Muzazu SGY Assefa DG Phiri C Getinet T Solomon S Yismaw G . Prevalence of cryptococcal meningitis among people living with human immunodeficiency virus and predictors of mortality in adults on induction therapy in Africa: A systematic review and meta-analysis. Front Med. (2022) 9:989265. doi: 10.3389/fmed.2022.989265, PMID: 36160163 PMC9494297

[9] Pasquier E Kunda J De Beaudrap P Loyse A Temfack E Molloy SF . Long-term mortality and disability in cryptococcal meningitis: A systematic literature review. Clin Infect Dis: an Off Publ Infect Dis Soc America. (2018) 66:1122–32. doi: 10.1093/cid/cix870, PMID: 29028957

[10] Alanio A . Dormancy in Cryptococcus neoformans: 60 years of accumulating evidence. J Clin Invest. (2020) 130:3353–60. doi: 10.1172/JCI136223, PMID: 32484459 PMC7324190

[11] Francis VI Liddle C Camacho E Kulkarni M Junior SRS Harvey JA . *Cryptococcus neoforman*s rapidly invades the murine brain by sequential breaching of airway and endothelial tissues barriers, followed by engulfment by microglia. mBio. (2024) 15:e0307823. doi: 10.1128/mbio.03078-23, PMID: 38511961 PMC11005363

[12] Mohamed SH Fu MS Hain S Alselami A Vanhoffelen E Li Y . Microglia are not protective against cryptococcal meningitis. Nat Commun. (2023) 14:7202. doi: 10.1038/s41467-023-43061-0, PMID: 37938547 PMC10632471

[13] Enriquez V Munzen ME Porras LM Charles-Niño CL Yu F Alviña K . Active Cryptococcus neoformans glucuronoxylomannan production prevents elimination of cryptococcal CNS infection in *vivo*. J Neuroinflamm. (2025) 22:61. doi: 10.1186/s12974-025-03384-9, PMID: 40038673 PMC11877788

[14] Barbosa FM Fonseca FL Holandino C Alviano CS Nimrichter L Rodrigues ML . Glucuronoxylomannan-mediated interaction of Cryptococcus neoformans with human alveolar cells results in fungal internalization and host cell damage. Microbes Infect. (2006) 8:493–502. doi: 10.1016/j.micinf.2005.07.027, PMID: 16293436

[15] McFadden D Zaragoza O Casadevall A . The capsular dynamics of Cryptococcus neoformans. Trends Microbiol. (2006) 14:497–505. doi: 10.1016/j.tim.2006.09.003, PMID: 16996739

[16] Lee SC Casadevall A Dickson DW . Immunohistochemical localization of capsular polysaccharide antigen in the central nervous system cells in cryptococcal meningoencephalitis. Am J Pathol. (1996) 148:1267–74., PMID: 8644867 PMC1861512

[17] Zaragoza O Rodrigues ML De Jesus M Frases S Dadachova E Casadevall A . The capsule of the fungal pathogen Cryptococcus neoformans. Adv Appl Microbiol. (2009) 68:133–216. doi: 10.1016/S0065-2164(09)01204-0, PMID: 19426855 PMC2739887

[18] Vecchiarelli A Pericolini E Gabrielli E Kenno S Perito S Cenci E . Elucidating the immunological function of the Cryptococcus neoformans capsule. Future Microbiol. (2013) 8:1107–16. doi: 10.2217/fmb.13.84, PMID: 24020739

[19] Colombo AC Rella A Normile T Joffe LS Tavares PM de S Araújo GR . *Cryptococcus neoformans* glucuronoxylomannan and sterylglucoside are required for host protection in an animal vaccination model. mBio. (2019) 10:e02909–18. doi: 10.1128/mBio.02909-18, PMID: 30940711 PMC6445945

[20] Hamed MF Enriquez V Munzen ME Charles-Niño CL Mihu MR Khoshbouei H . Clinical and pathological characterization of Central Nervous System cryptococcosis in an experimental mouse model of stereotaxic intracerebral infection. PloS Negl Trop Dis. (2023) 17:e0011068. doi: 10.1371/journal.pntd.0011068, PMID: 36656900 PMC9888703

[21] Lee HH Carmichael DJ Ríbeiro V Parisi DN Munzen ME Charles-Niño CL . Glucuronoxylomannan intranasal challenge prior to Cryptococcus neoformans pulmonary infection enhances cerebral cryptococcosis in rodents. PloS Pathog. (2023) 19:e1010941. doi: 10.1371/journal.ppat.1010941, PMID: 37115795 PMC10171644

[22] Muchmore HG Scott EN Felton FG Fromtling RA . Cryptococcal capsular polysaccharide clearance in nonimmune mice. Mycopathologia. (1982) 78:41–5. doi: 10.1007/BF00436580, PMID: 7048100

[23] Lu H Zhou Y Yin Y Pan X Weng X . Cryptococcal antigen test revisited: significance for cryptococcal meningitis therapy monitoring in a tertiary chinese hospital. J Clin Microbiol. (2005) 43:2989–90. doi: 10.1128/JCM.43.6.2989-2990.2005, PMID: 15956440 PMC1151949

[24] Bennett JE Williamson PR . Antigen titers in cryptococcal meningitis: what determines how fast they fall? J Infect Dis. (2024) 230:1291–6. doi: 10.1093/infdis/jiae354, PMID: 38986025 PMC11566034

[25] Qian X Nguyen HN Song MM Hadiono C Ogden SC Hammack C . Brain-region-specific organoids using mini-bioreactors for modeling ZIKV exposure. Cell. (2016) 165:1238–54. doi: 10.1016/j.cell.2016.04.032, PMID: 27118425 PMC4900885

[26] Gabriel E Ramani A Karow U Gottardo M Natarajan K Gooi LM . Recent zika virus isolates induce premature differentiation of neural progenitors in human brain organoids. Cell Stem Cell. (2017) 20:397–406.e5. doi: 10.1016/j.stem.2016.12.005, PMID: 28132835

[27] D’Aiuto L Bloom DC Naciri JN Smith A Edwards TG McClain L . Modeling herpes simplex virus 1 infections in human central nervous system neuronal cells using two- and three-dimensional cultures derived from induced pluripotent stem cells. J Virol. (2019) 93:e00111–19. doi: 10.1128/JVI.00111-19, PMID: 30787148 PMC6475775

[28] Dos Reis RS Sant S Keeney H Wagner MCE Ayyavoo V . Modeling HIV-1 neuropathogenesis using three-dimensional human brain organoids (hBORGs) with HIV-1 infected microglia. Sci Rep. (2020) 10:15209. doi: 10.1038/s41598-020-72214-0, PMID: 32938988 PMC7494890

[29] Sun G Chiuppesi F Chen X Wang C Tian E Nguyen J . Modeling human cytomegalovirus-induced microcephaly in human iPSC-derived brain organoids. Cell Rep Med. (2020) 1:100002. doi: 10.1016/j.xcrm.2020.100002, PMID: 33205055 PMC7659592

[30] Lancaster MA Knoblich JA . Generation of cerebral organoids from human pluripotent stem cells. Nat Protoc. (2014) 9:2329–40. doi: 10.1038/nprot.2014.158, PMID: 25188634 PMC4160653

[31] Ormel PR Vieira de Sá R van Bodegraven EJ Karst H Harschnitz O Sneeboer MAM . Microglia innately develop within cerebral organoids. Nat Commun. (2018) 9:4167. doi: 10.1038/s41467-018-06684-2, PMID: 30301888 PMC6177485

[32] Cherniak R Morris LC Anderson BC Meyer SA . Facilitated isolation, purification, and analysis of glucuronoxylomannan of Cryptococcus neoformans. Infect Immun. (1991) 59:59–64. doi: 10.1128/iai.59.1.59-64.1991, PMID: 1987064 PMC257705

[33] Wozniak KL Levitz SM . Isolation and purification of antigenic components of Cryptococcus. Methods Mol Biol (Clifton NJ). (2009) 470:71–83. doi: 10.1007/978-1-59745-204-5_7, PMID: 19089377 PMC3297022

[34] Ballal AR Reddy SK Chandran D Hegde S Upadhya R Se PK . Cell-specific extracellular vesicle-encapsulated exogenous GABA controls seizures in epilepsy. Stem Cell Res Therapy. (2024) 15:108. doi: 10.1186/s13287-024-03721-4, PMID: 38637847 PMC11027552

[35] Reddy SK Ballal AR Shailaja S Seetharam RN Raghu CH Sankhe R . Small extracellular vesicle-loaded bevacizumab reduces the frequency of intravitreal injection required for diabetic retinopathy. Theranostics. (2023) 13:2241–55. doi: 10.7150/thno.78426, PMID: 37153730 PMC10157735

[36] Bligh EG Dyer WJ . A rapid method of total lipid extraction and purification. Can J Biochem Physiol. (1959) 37:911–7. doi: 10.1139/o59-099, PMID: 13671378

[37] Chakraborty A Hegde S Praharaj SK Prabhu K Patole C Shetty AK . Age related prevalence of mild cognitive impairment in type 2 diabetes mellitus patients in the Indian population and association of serum lipids with cognitive dysfunction. Front Endocrinol (Lausanne. (2021) 12:798652. doi: 10.3389/fendo.2021.798652, PMID: 35035379 PMC8758578

[38] Lu C Wu C Ghoreishi D Chen W Wang L Damm W . OPLS4: improving force field accuracy on challenging regimes of chemical space. J Chem Theory Comput. (2021) 17:4291–300. doi: 10.1021/acs.jctc.1c00302, PMID: 34096718

[39] Bowers KJ Chow DE Xu H Dror RO Eastwood MP Gregersen BA . (2006). Scalable algorithms for molecular dynamics simulations on commodity clusters, in: SC ‘06: Proceedings of the 2006 ACM/IEEE Conference on Supercomputing, Tampa, FL, USA, Vol. 2006. pp. 43–3. doi: 10.1109/SC.2006.54

[40] Kuttel MM Casadevall A Oscarson S . *Cryptococcus neoformans* capsular GXM conformation and epitope presentation: A molecular modelling study. Mol (Basel Switzerland). (2020) 25:2651. doi: 10.3390/molecules25112651, PMID: 32517333 PMC7321252

[41] Xu J Ganguly A Zhao J Ivey M Lopez R Osterholzer JJ . CCR2 signaling promotes brain infiltration of inflammatory monocytes and contributes to neuropathology during cryptococcal meningoencephalitis. mBio. (2021) 12:e0107621. doi: 10.1128/mBio.01076-21, PMID: 34311579 PMC8406332

[42] Gasparovic C Rosenberg GA Wallace JA Estrada EY Roberts K Pastuszyn A . Magnetic resonance lipid signals in rat brain after experimental stroke correlate with neutral lipid accumulation. Neurosci Lett. (2001) 301:87–90. doi: 10.1016/s0304-3940(01)01616-0, PMID: 11248429

[43] Listenberger LL Han X Lewis SE Cases S Farese RV Ory DS . Triglyceride accumulation protects against fatty acid-induced lipotoxicity. Proc Natl Acad Sci United States America. (2003) 100:3077–82. doi: 10.1073/pnas.0630588100, PMID: 12629214 PMC152249

[44] Martínez L Andrade R Birgin EG Martínez JM . PACKMOL: a package for building initial configurations for molecular dynamics simulations. J Comput Chem. (2009) 30:2157–64. doi: 10.1002/jcc.21224, PMID: 19229944

[45] Schrödinger . Materials science suite. Schrödinger Release 2024-4. (2024) Available online at: https://www.schrodinger.com/life-science/download/release-notes/release-2024-4/ (Accessed July 10, 2025).

[46] Lendvai N Casadevall A Liang Z Goldman DL Mukherjee J Zuckier L . Effect of immune mechanisms on the pharmacokinetics and organ distribution of cryptococcal polysaccharide. J Infect Dis. (1998) 177:1647–59. doi: 10.1086/515329, PMID: 9607845

[47] Grinsell M Weinhold LC Cutler JE Han Y Kozel TR . *In vivo* clearance of glucuronoxylomannan, the major capsular polysaccharide of Cryptococcus neoformans: a critical role for tissue macrophages. J Infect Dis. (2001) 184:479–87. doi: 10.1086/322787, PMID: 11471106

[48] Chiapello LS Baronetti JL Garro AP Spesso MF Masih DT . Cryptococcus neoformans glucuronoxylomannan induces macrophage apoptosis mediated by nitric oxide in a caspase-independent pathway. Int Immunol. (2008) 20:1527–41. doi: 10.1093/intimm/dxn112, PMID: 18927317

[49] Villena SN Pinheiro RO Pinheiro CS Nunes MP Takiya CM DosReis GA . Capsular polysaccharides galactoxylomannan and glucuronoxylomannan from Cryptococcus neoformans induce macrophage apoptosis mediated by Fas ligand. Cell Microbiol. (2008) 10:1274–85. doi: 10.1111/j.1462-5822.2008.01125.x, PMID: 18284419

[50] Rodrigues ML Fonseca FL Frases S Casadevall A Nimrichter L . The still obscure attributes of cryptococcal glucuronoxylomannan. Med Mycol. (2009) 47:783–8. doi: 10.3109/13693780902788621, PMID: 19343609 PMC4318802

[51] Chang ZL Netski D Thorkildson P Kozel TR . Binding and internalization of glucuronoxylomannan, the major capsular polysaccharide of Cryptococcus neoformans, by murine peritoneal macrophages. Infect Immun. (2006) 74:144–51. doi: 10.1128/IAI.74.1.144-151.2006, PMID: 16368967 PMC1346664

[52] Monari C Retini C Casadevall A Netski D Bistoni F Kozel TR . Differences in outcome of the interaction between Cryptococcus neoformans glucuronoxylomannan and human monocytes and neutrophils. Eur J Immunol. (2003) 33:1041–51. doi: 10.1002/eji.200323388, PMID: 12672070

[53] Hernández-Chávez MJ Pérez-García LA Niño-Vega GA Mora-Montes HM . Fungal strategies to evade the host immune recognition. J Fungi. (2017) 3:51. doi: 10.3390/jof3040051, PMID: 29371567 PMC5753153

[54] García-Carnero LC Gómez-Gaviria M Tamez-Castrellón AK Mora-Montes HM . Host immune responses to fungal infection. In: Molecular Medical Microbiology (Third Edition). Academic press. (2024). p. 2823–46.

[55] Yauch LE Mansour MK Shoham S Rottman JB Levitz SM . Involvement of CD14, toll-like receptors 2 and 4, and MyD88 in the host response to the fungal pathogen Cryptococcus neoformans in *vivo*. Infect Immun. (2004) 72:5373–82. doi: 10.1128/IAI.72.9.5373-5382.2004, PMID: 15322035 PMC517466

[56] Biondo C Midiri A Messina L Tomasello F Garufi G Catania MR . MyD88 and TLR2, but not TLR4, are required for host defense against Cryptococcus neoformans. Eur J Immunol. (2005) 35:870–8. doi: 10.1002/eji.200425799, PMID: 15714580

[57] Olave MC Vargas-Zambrano JC Celis AM Castañeda E González JM . Infective capacity of Cryptococcus neoformans and Cryptococcus gattii in a human astrocytoma cell line. Mycoses. (2017) 60:447–53. doi: 10.1111/myc.12619, PMID: 28338245

[58] Goldman DL Casadevall A Cho Y Lee SC . Cryptococcus neoformans meningitis in the rat. Lab Invest J Tech Methods Pathol. (1996) 75:759–70. 8973471

[59] Traino K Snow J Ham L Summers A Segalà L Shirazi T . HIV-negative cryptococcal meningoencephalitis results in a persistent frontal-subcortical syndrome. Sci Rep. (2019) 9:18442. doi: 10.1038/s41598-019-54876-7, PMID: 31804566 PMC6895107

[60] Liu Y Fan L Jiang X Lu Y Li Y . A case study of repetitive transcranial magnetic stimulation for cryptococcal meningitis combined with cognitive impairment. Front Hum Neurosci. (2022) 16:1061916. doi: 10.3389/fnhum.2022.1061916, PMID: 36590060 PMC9800931

[61] Chen SC Slavin MA Heath CH Playford EG Byth K Marriott D . Clinical manifestations of Cryptococcus gattii infection: determinants of neurological sequelae and death. Clin Infect Dis: an Off Publ Infect Dis Soc America. (2012) 55:789–98. doi: 10.1093/cid/cis529, PMID: 22670042

[62] Lu CH Chen HL Chang WN Tsai NW Wang HC Yang TM . Assessing the chronic neuropsychologic sequelae of human immunodeficiency virus-negative cryptococcal meningitis by using diffusion tensor imaging. AJNR Am J Neuroradiol. (2011) 32:1333–9. doi: 10.3174/ajnr.A2489, PMID: 21596808 PMC7966046

[63] Chen MH Lu CH Wang HC Chen HL Tsai NW Li SH . Long-term neuropsychological sequelae in HIV-seronegative cryptococcal meningoencephalitis patients with and without ventriculoperitoneal shunts: a cine MRI study. Behav Neurol. (2015) 2015:356476. doi: 10.1155/2015/356476, PMID: 25948879 PMC4407401

[64] Frick KM Fernandez SM . Enrichment enhances spatial memory and increases synaptophysin levels in aged female mice. Neurobiol Aging. (2003) 24:615–26. doi: 10.1016/s0197-4580(02)00138-0, PMID: 12714119

[65] Birch AM McGarry NB Kelly AM . Short-term environmental enrichment, in the absence of exercise, improves memory, and increases NGF concentration, early neuronal survival, and synaptogenesis in the dentate gyrus in a time-dependent manner. Hippocampus. (2013) 23:437–50. doi: 10.1002/hipo.22103, PMID: 23460346

[66] Lahiri DK Ge YW Farlow MR . Effect of a memory-enhancing drug, AIT-082, on the level of synaptophysin. Ann New York Acad Sci. (2000) 903:387–93. doi: 10.1111/j.1749-6632.2000.tb06390.x, PMID: 10818529

[67] Park SW Lee CH Cho HY Seo MK Lee JG Lee BJ . Effects of antipsychotic drugs on the expression of synaptic proteins and dendritic outgrowth in hippocampal neuronal cultures. Synapse (New York NY). (2013) 67:224–34. doi: 10.1002/syn.21634, PMID: 23335099

[68] Seo MK Lee CH Cho HY Lee JG Lee BJ Kim JE . Effects of antidepressant drugs on synaptic protein levels and dendritic outgrowth in hippocampal neuronal cultures. Neuropharmacology. (2014) 79:222–33. doi: 10.1016/j.neuropharm.2013.11.019, PMID: 24296153

[69] Seo MK Lee CH Cho HY You YS Lee BJ Lee JG . Effects of antipsychotic drugs on the expression of synapse-associated proteins in the frontal cortex of rats subjected to immobilization stress. Psychiatry Res. (2015) 229:968–74. doi: 10.1016/j.psychres.2015.05.098, PMID: 26254796

[70] van der Meer-Janssen YP van Galen J Batenburg JJ Helms JB . Lipids in host-pathogen interactions: pathogens exploit the complexity of the host cell lipidome. Prog Lipid Res. (2010) 49:1–26. doi: 10.1016/j.plipres.2009.07.003, PMID: 19638285 PMC7112618

[71] Lee WJ Ryu YJ Moon J Lee ST Jung KH Park KI . Enlarged periventricular space and periventricular lesion extension on baseline brain MRI predicts poor neurological outcomes in cryptococcus meningoencephalitis. Sci Rep. (2021) 11:6446. doi: 10.1038/s41598-021-85998-6, PMID: 33742090 PMC7979784

[72] Meyerhoff DJ Bloomer C Cardenas V Norman D Weiner MW Fein G . Elevated subcortical choline metabolites in cognitively and clinically asymptomatic HIV+ patients. Neurology. (1999) 52:995–1003. doi: 10.1212/wnl.52.5.995, PMID: 10102419

[73] Tracey I Carr CA Guimaraes AR Worth JL Navia BA González RG . Brain choline-containing compounds are elevated in HIV-positive patients before the onset of AIDS dementia complex: A proton magnetic resonance spectroscopic study. Neurology. (1996) 46:783–8. doi: 10.1212/wnl.46.3.783, PMID: 8618683

[74] Fitzner D Bader JM Penkert H Bergner CG Su M Weil MT . Cell-type- and brain-region-resolved mouse brain lipidome. Cell Rep. (2020) 32:108132. doi: 10.1016/j.celrep.2020.108132, PMID: 32937123

[75] Neumann EK Comi TJ Rubakhin SS Sweedler JV . Lipid heterogeneity between astrocytes and neurons revealed by single-cell MALDI-MS combined with immunocytochemical classification. Angewandte Chemie (International Ed English). (2019) 58:5910–4. doi: 10.1002/anie.201812892, PMID: 30811763 PMC6461516

[76] Roy P Tomassoni D Nittari G Traini E Amenta F . Effects of choline containing phospholipids on the neurovascular unit: A review. Front Cell Neurosci. (2022) 16:988759. doi: 10.3389/fncel.2022.988759, PMID: 36212684 PMC9541750

[77] Chrisman CJ Albuquerque P Guimaraes AJ Nieves E Casadevall A . Phospholipids trigger Cryptococcus neoformans capsular enlargement during interactions with amoebae and macrophages. PloS Pathog. (2011) 7:e1002047. doi: 10.1371/journal.ppat.1002047, PMID: 21637814 PMC3102711

[78] Cherniak R Sundstrom JB . Polysaccharide antigens of the capsule of Cryptococcus neoformans. Infect Immun. (1994) 62:1507–12. doi: 10.1128/iai.62.5.1507-1512.1994, PMID: 8168912 PMC186341

[79] Frases S Nimrichter L Viana NB Nakouzi A Casadevall A . *Cryptococcus neoformans* capsular polysaccharide and exopolysaccharide fractions manifest physical, chemical, and antigenic differences. Eukaryotic Cell. (2008) 7:319–27. doi: 10.1128/EC.00378-07, PMID: 18156290 PMC2238165

[80] Fonseca FL Frases S Casadevall A Fischman-Gompertz O Nimrichter L Rodrigues ML . Structural and functional properties of the *Trichosporon asahii* glucuronoxylomannan. Fungal Genet Biol: FG B. (2009) 46:496–505. doi: 10.1016/j.fgb.2009.03.003, PMID: 19285564 PMC4318814

[81] Litvintseva AP Lin X Templeton I Heitman J Mitchell TG . Many globally isolated AD hybrid strains of Cryptococcus neoformans originated in Africa. PloS Pathog. (2007) 3:e114. doi: 10.1371/journal.ppat.0030114, PMID: 17708680 PMC1949410

[82] Desnos-Ollivier M Patel S Raoux-Barbot D Heitman J Dromer F French Cryptococcosis Study Group . Cryptococcosis serotypes impact outcome and provide evidence of cryptococcus neoformans speciation. mBio. (2015) 6:e00311. doi: 10.1128/mBio.00311-15, PMID: 26060271 PMC4462623

