# Supplementary material for: Host-membrane lipid composition controls Cryptococcus neoformans cellular targets
Source: Front Immunol. 2026 Jan 6;16:1687068. doi: 10.3389/fimmu.2025.1687068 (PMC12815873; doi:10.3389/fimmu.2025.1687068)
Supplement: Supplementary file 1 [file Presentation1.pptx]

## Slide 1
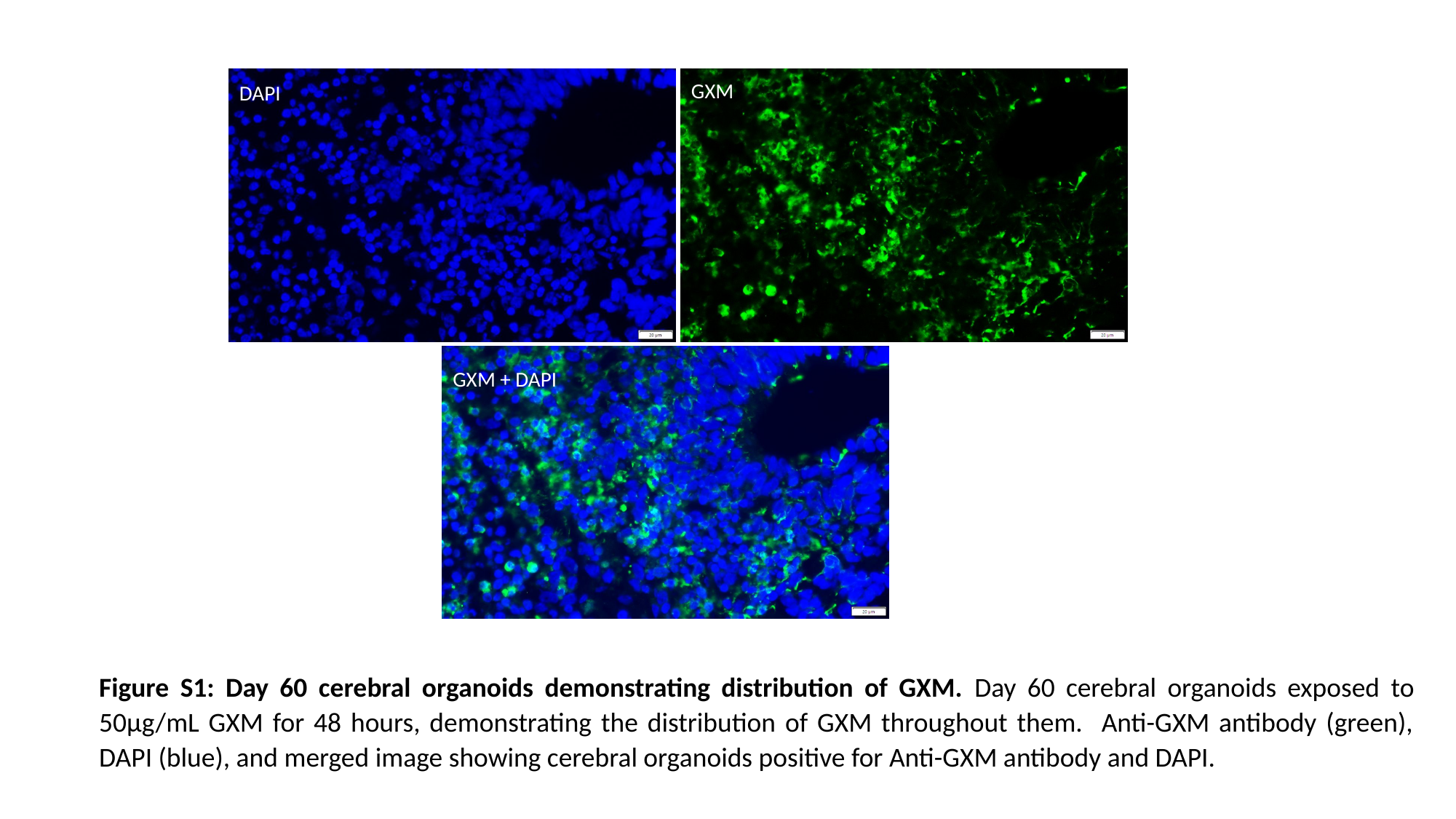

DAPI
GXM
GXM + DAPI
Figure S1: Day 60 cerebral organoids demonstrating distribution of GXM. Day 60 cerebral organoids exposed to 50µg/mL GXM for 48 hours, demonstrating the distribution of GXM throughout them. Anti-GXM antibody (green), DAPI (blue), and merged image showing cerebral organoids positive for Anti-GXM antibody and DAPI.
