# Supplementary material for: Host-membrane lipid composition controls Cryptococcus neoformans cellular targets
Source: Front Immunol. 2026 Jan 6;16:1687068. doi: 10.3389/fimmu.2025.1687068 (PMC12815873; doi:10.3389/fimmu.2025.1687068)
Supplement: Supplementary file 2 [file Table1.docx]

**Supplementary Table 1**

| **REAGENT or RESOURCE** | **SOURCE** | **IDENTIFIER** |
| --- | --- | --- |
| **Antibodies** | | |
| SOX2 monoclonal antibody (1:200) | Invitrogen | MA531455 |
| NESTIN polyclonal antibody (1:250) | Invitrogen | PA585757 |
| TUJ1 polyclonal antibody (1:500) | Merk | T2200 |
| Ki67 monoclonal antibody (1:500) | Invitrogen | MA514520 |
| GFAP monoclonal antibody (1:250) | Invitrogen | 14989282 |
| Anti-Glucuronoxylomannan (GXM) antibody, clone 18B7 (1:200) | Sigma | MABF2069 |
| Synaptophysin monoclonal antibody (1:200) | ThermoFisher | MA514532 |
| IBA 1 Polyclonal antibody (1:200) | Elabsciences | E-AB- 10382 |
| SOX 10 Polyclonal Antibody (1:200) | Invitrogen | PA5-40697 |
| Goat anti-Rabbit IgG (H+L) Cross-Adsorbed Secondary Antibody, HRP (1: 1000) | Invitrogen | G21234 |
| Goat anti-Mouse IgG (H+L) Cross-Adsorbed Secondary Antibody, HRP (1: 1000) | Invitrogen | G21040 |
| Donkey anti-Mouse IgG (H+L) ReadyProbes™ Secondary Antibody, Alexa Fluor™ 488 (1: 250) | ThermoFisher | R37114 |
| Donkey anti-Rabbit IgG (H+L) Highly Cross-Adsorbed Secondary Antibody, Alexa Fluor™ 568 (1: 250) | ThermoFisher | A10042 |
|  |  |  |
| **Fungal strains** | | |
| *Cryptococcus neoformans* | ATCC | ATCC 32045 |
|  |  |  |
| **Chemicals, peptides, and recombinant proteins** | | |
| DMEM/F12 medium | ThermoFisher | 11330032 |
| Neurobasal plus Medium | ThermoFisher | A3582901 |
| StemFlex medium | Gibco | A3349401 |
| PSC Neural Induction Medium | Gibco | A1647801 |
| Matrigel Growth Factor Reduced (GFR) Basement Membrane Matrix, | Corning | 354230 |
| Dispase II | Sigma | D4693 |
| GlutaMAX™ Supplement | ThermoFisher | 35050-061 |
| MEM Non-Essential Amino Acids Solution (100X) | ThermoFisher | 11140050 |
| 2-Mercaptoethanol | Sigma | M3148-25ML |
| Heparin sodium salt | Himedia | RM554 |
| ROCK inhibitor (Y-27632) | Pepro Tech | SM- 1293823 |
| N-2 Supplement (100X) | ThermoFisher | 17502048 |
| B-27™ Supplement (50X), minus vitamin A | ThermoFisher | 12587010 |
| B-27™ Plus Supplement (50X) | ThermoFisher | A3582801 |
| Penicillin-Streptomycin (10,000 U/mL) | Thermo Scientific | 15140122 |
| Insulin solution human | Sigma | I9278-5ML |
| Poly-L-ornithine solution | Sigma | P4957 |
| Laminin Mouse Protein, Natural | Gibco | 23017015 |
| BDNF, Human | Genscript | Z03208-5 |
| GDNF, Human | Genscript | Z03387-10 |
| YPD growth medium | Himedia | G037-500G |
| DPBS, no calcium, no magnesium | ThermoFisher | 14190144 |
| Sodium acetate anhydrous | Loba | 05755 00250 |
| Ethyl alcohol | Heyman | F204325 |
| Acetic acid | ESSARKAY | A74229 |
| NaCl | Finar | 11570SR500 |
| CTAB | Loba | 268000500 |
| Phenol | Loba | 516800100 |
| Sulfuric acid | Loba | 002902500M |
| Dextrose anhydrous | Loba | 317000500 |
| Triton X 100 | Himedia | MB031-100ml |
| DAPI dihydrochloride | Himedia | MBO97-10mg |
| Donkey serum | Merck | S30-100ml |
| Paraformaldehyde | Himedia | GRM3660-500G |
| Gelatin type A | Himedia | MB169-500g |
| Sucrose | Himedia | GRM3063-500G |
| Chloroform | Himedia | AS114-1L |
| Methanol | Finar | 40930LC250 |
| RIPA Buffer | Sigma | R0278-50m |
| Protease inhibitor | Sigma | P8340-1ML |
| Acrylamide/Bis-acrylamide solution 30% (29:1) | Himedia | ML037-500ML |
| Tris Buffer | SRL | 71033 |
| SDS | Himedia | Mb010_100 |
| Ammonium Persulphate | Biorad | 16010700 |
| TEMED | Himedia | MBO26-100ml |
| Isopropyl alcohol | Loba | 0273D 0025K |
| 2X Laemmli Buffer | Himedia | ML021-6ML |
| Glycine | Himedia | MB013-500G |
| Acetic acid | ESSARKAY | A74229 |
| Tween 20 | Himedia | MB067-100ML |
| Non-fat Milk powder | Sagar | N/A |
|  |  |  |
| **Commercial kits and enzymes** | | |
| TUNEL Assay Kit | TAKARA | MK 500 |
| Pierce BCA protein assay kit | Thermo Scientific | 23227 |
| **Experimental models: Cell lines** | | |
| Human induced pluripotent stem cells | National Institutes of Health - Center for Regenerative Medicine (CRM) | ND1.4, RRID:CVCL_1E77 |
|  |  |  |
| **Other** | | |
| PAP pen | Rayudu laboratories Ltd | N/A |
| Slide-A-Lyser Dialysis Cassettes | Invitrogen | 66380 |
| Parafilm | Abdos | U30102 |
| Cell culture plate (6 well) | Himedia | TPP6 |
| Cell culture plate (12 well) | Abdos | P21021 |
| Cell culture plate (24 well) | Himedia | TPP24 |
| Coverslip 15mm sterile | Himedia | GRM3063-500G |
| Hi-FiBloE™ PVDF Membrane for Blotting Pore Size: 0.2 μm Dimensions: 26 cm X 3.3 m | Himedia | MBM004-1RL |
